# Galantamine prevents and reverses neuroimmune induction and loss of adult hippocampal neurogenesis following adolescent alcohol exposure

**DOI:** 10.1186/s12974-021-02243-7

**Published:** 2021-09-16

**Authors:** Victoria Macht, Ryan Vetreno, Natalie Elchert, Fulton Crews

**Affiliations:** 1 Bowles Center for Alcohol Studies, School of Medicine, University of North Carolina at Chapel Hill, 104 Manning Drive, Chapel Hill, NC 27599 USA; 2 Department of Psychiatry, School of Medicine, University of North Carolina at Chapel Hill, Chapel Hill, NC USA; 3 Department of Pharmacology, School of Medicine, University of North Carolina at Chapel Hill, Chapel Hill, NC USA

**Keywords:** Proinflammatory, HMGB1, Doublecortin, Cyclooxygenase, Neuroprogenitor, Cell death, Alcohol

## Abstract

**Background:**

Binge ethanol exposure during adolescence reduces hippocampal neurogenesis, a reduction which persists throughout adulthood despite abstinence. This loss of neurogenesis, indicated by reduced doublecortin+ immunoreactivity (DCX+IR), is paralleled by an increase in hippocampal proinflammatory signaling cascades. As galantamine, a cholinesterase inhibitor, has anti-inflammatory actions, we tested the hypothesis that galantamine would prevent (study 1) or restore (study 2) AIE induction of proinflammatory signals within the hippocampus as well as AIE-induced loss of hippocampal neurogenesis.

**Methods:**

Galantamine (4 mg/kg) or vehicle (saline) was administered to Wistar rats during adolescent intermittent ethanol (AIE; 5.0 g/kg ethanol, 2 days on/2 days off, postnatal day [P] 25-54) (study 1, prevention) or after AIE during abstinent maturation to adulthood (study 2, restoration).

**Results:**

Results indicate AIE reduced DCX+IR and induced cleaved caspase3 (Casp3) in DCX-expressing immature neurons. Excitingly, AIE induction of activated Casp3 in DCX-expressing neurons is both prevented and reversed by galantamine treatment, which also resulted in prevention and restoration of neurogenesis (DCX+IR). Similarly, galantamine prevented and/or reversed AIE induction of proinflammatory markers, including the chemokine (C-C motif) ligand 2 (CCL2), cyclooxygenase-2 (COX-2), and high mobility group box 1 (HMGB1) protein, suggesting that AIE induction of proinflammatory signaling mediates both cell death cascades and hippocampal neurogenesis. Interestingly, galantamine treatment increased Ki67+IR generally as well as increased pan-Trk expression specifically in AIE-treated rats but failed to reverse AIE induction of NADPH-oxidase (gp91^phox^).

**Conclusions:**

Collectively, our studies suggest that (1) loss of neurogenesis after AIE is mediated by persistent induction of proinflammatory cascades which drive activation of cell death machinery in immature neurons, and (2) galantamine can prevent and restore AIE disruptions in the hippocampal environmental milieu to then prevent and restore AIE-mediated loss of neurogenesis.

**Supplementary Information:**

The online version contains supplementary material available at 10.1186/s12974-021-02243-7.

## Background

Adolescence is a period of robust hippocampal neurogenesis where new neurons are functionally integrated into the granule cell layer and influence learning and memory processes [1]. The birth, differentiation, integration, and selective apoptosis of these new neurons is maintained via a delicate balance within the neurogenic niche, requiring coordination between neurons and glia to mediate the production of growth factors and proinflammatory signals [2]. Moreover, it is becoming increasingly appreciated that neurogenic disruptions during adolescence (e.g., binge ethanol exposure) persist throughout adulthood [3] and are driven by upregulation of proinflammatory factors paralleled by a loss of trophic support (for review see [2]) as well as dysregulation of cholinergic anti-inflammatory networks [4, 5]. As neurogenic deficits are a hallmark feature of neurodegenerative diseases [6, 7], this suggests that insults during adolescence may create an accelerated track for later neurocognitive decline by disrupting healthy neural physiology toward one with a proinflammatory imbalance, resulting in long-term deficits in neurogenesis. Consequently, understanding factors which can prevent or rescue developmental deficits in hippocampal neurogenesis is a cornerstone to understanding the balance between neuronal health and disease. 

Preclinical models indicate that ethanol dramatically inhibits adult and adolescent hippocampal neurogenesis with effects evident after acute [8] and chronic intake [9, 10]. Effects of adolescent intermittent ethanol (AIE) exposure across puberty are particularly devastating, leading to long-lasting losses in adult hippocampal neurogenesis, whereas identical adult exposure only transiently reduces neurogenesis [3]. Interestingly, prior studies by Crews et al. [8] and Morris et al. [11] indicate that adolescent ethanol exposure decreases proliferation/survival of hippocampal progenitor cells which mostly (< 90%) become neurons [8]. Therefore, the current study used doublecortin (DCX), which marks immature neurons, to examine the effects of adolescent ethanol exposure on hippocampal neurogenesis rather than gliogenesis.

One of the mechanisms thought to underlie these persistent deficits in neurogenesis after AIE is a continuous disruption of the environmental milieu within the neurogenic niche toward a proinflammatory state, resulting in impaired survival of newborn neurons. In support of this concept, several studies have indicated that AIE increases hippocampal neuroinflammatory cascades also evident in other neurodegenerative diseases, including high mobility group box 1 (HMGB1) protein, an agonist at the receptor for advanced glycation end products (RAGE), and activation of the proinflammatory transcription factor nuclear factor kappa-light-chain enhancer of activated B cells (NF-κB) [2, 4, 5, 12]. These persistent neurogenic deficits after AIE are accompanied by cognitive dysfunction in hippocampal-dependent spatial tasks such as the Morris water maze [13] and the Barnes maze [14, 15], emphasizing that ethanol-induced deficits in neurogenesis and induction of proinflammatory cascades within the hippocampus are tightly coupled to cognitive function.

Luckily, emerging evidence suggests that this induction of proinflammatory cascades and reduction in neurogenesis after AIE are both reversible [5], indicating plasticity within hippocampal circuitry. For example, exercise can prevent and reverse ethanol’s induction of proinflammatory genes and reduction of neurogenesis to restore AIE-induced cognitive dysfunction in hippocampal-dependent spatial tasks such as the Morris water maze [5]. Similarly, the non-steroidal anti-inflammatory drug indomethacin can prevent ethanol-induced deficits in hippocampal neurogenesis [10]. Collectively, these results emphasize that neuroinflammation is a reversible mediator of adolescent ethanol-induced deficits in hippocampal neurogenesis and cognitive dysfunction, potentially impacting subsequent neurodegenerative disease susceptibility. However, the exact mechanism underlying AIE’s induction of hippocampal proinflammatory cascades and impairment of neurogenesis remains an important area of investigation.

In many neurodegenerative diseases, increased neuroinflammation is paralleled by a reduction in cholinergic modulation [16]. This is unsurprising as cholinergic systems play important inhibitory regulatory roles for both systemic monocytes and proinflammatory cytokines [17] as well as regulating proinflammatory gene expression in the central nervous system (CNS) [18]. Within the CNS, neurons in the medial septum of the basal forebrain contain a subgroup of cholinergic neurons which primarily project to and regulate activity within the hippocampus. We have previously demonstrated that AIE significantly reduces medial septum basal forebrain cholinergic neurons [4, 5]. Loss of these cholinergic projections after AIE could play a role in hippocampal induction of inflammatory responses due to loss of acetylcholine activation of nicotinic and muscarinic receptors on both neurons and glia. Recent evidence suggests that AIE-induced loss of expression of cholinergic markers in the basal forebrain can be reversed by chronic treatment with galantamine [19]. These findings support the hypothesis that AIE-induced loss of cholinergic anti-inflammatory signaling contributes to the induction of hippocampal pro-inflammatory genes and the consequential loss of hippocampal neurogenesis. To test this hypothesis, the current study investigated whether chronic administration of the cholinesterase inhibitor galantamine can (1) prevent or (2) restore AIE-induced reductions in both hippocampal neurogenesis and proinflammatory markers.

## Materials and methods

### Animals

**Fig. 1:**
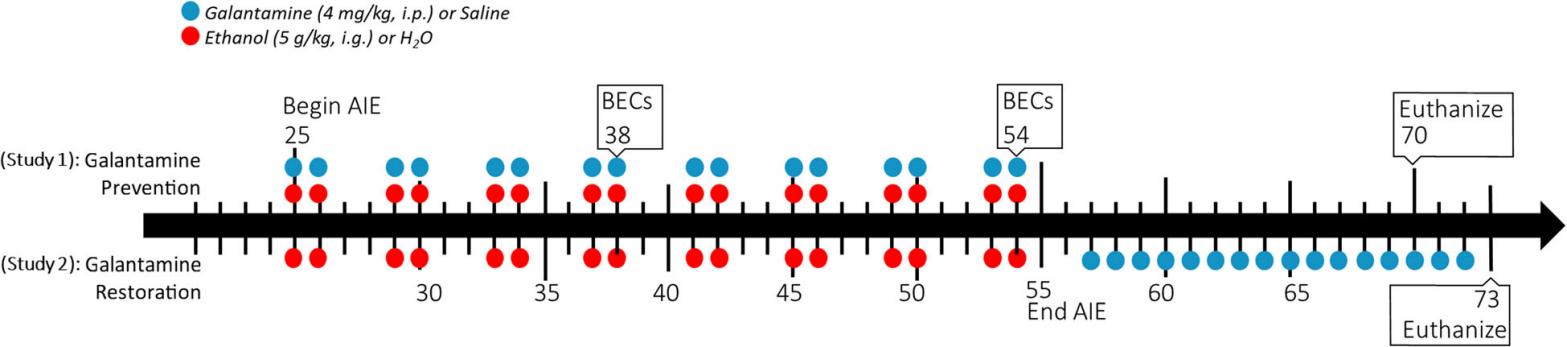
Experimental timeline. Two separate experiments were conducted to test whether galantamine can (1) prevent or (2) reverse AIE-induced deficits in hippocampal pathology. These studies were conducted in male Wistar rats. Between P25 and 54 in both studies, rats underwent a 2-day on/2-day off schedule of intragastric gavage with either 5 g/kg ethanol (AIE) or water (CON). In the prevention study (study 1, upper timeline), 4 mg/kg galantamine (Gal) (indicated by upper clue dots) or saline (Veh) was administered intraperitoneally as a pretreatment 30 min prior to ethanol/water gavage (indicated by upper red dots), i.e., 16 doses across 30 days. Treatment ended on P54 and rats were allowed to rest in their home cage until P70, after which they were euthanized and their brains harvested for IHC. In the restoration study (study 2, bottom timeline), rats completed the AIE paradigm (indicated by lower red dots) and then starting on P57 were administered intraperitoneal injections of either Gal or Veh (indicated by lower blue dots). Rats underwent 14 daily injections and then were euthanized on P73 when their brains were harvested for either IHC or western blotting (WB), respectively. In study 1, *n* = 42. In study 2, *n* = 39 for IHC, *n* = 29 for WB

Wistar rats were bred at the University of North Carolina at Chapel Hill in a temperature- (20 °C) and humidity-controlled vivarium on a 12/12 h light/dark cycle with lights on at 7:00 a.m. On the first postnatal day (P), litters were culled to 6 males and 4 females whenever possible. Males were weaned on P21 (± 2) and pair-housed according to treatment group using a split-litter design. Two different studies were performed to assess whether galantamine could either prevent (study 1, prevention) or reverse (study 2, restoration) AIE-induced pathogenesis in the hippocampus (Fig. 1). As such, each study had four different treatment groups: adolescent intermittent water control (CON)-Vehicle (CON-Veh), CON-Galantamine (CON-Gal), AIE-Veh, and AIE-Gal. See Table 1 for respective *n* sizes. All rats had *ad libitum* access to food and water throughout the experiment, and both studies were conducted in accordance with NIH regulations and with approval of the Institutional Animal Care and Use Committee at the University of North Carolina at Chapel Hill.

**Table 1:** Samples sizes by treatment group

	Vehicle	Galantamine
**CON**	Study 1: *n* = 9 (IHC)	Study 1: *n* = 12 (IHC)
	Study 2: *n* = 10 (IHC), 8 (WB)	Study 2: *n* = 11 (IHC), 8 (WB)
**AIE**	Study 1: *n* = 9 (IHC)	Study 1: *n* = 12 (IHC)
	Study 2: *n* = 9 (IHC), 6 (WB)	Study 2: *n* = 9 (IHC), 7 (WB)

*Immunohistochemistry (IHC), western blot (WB)

### AIE paradigm

Adolescent alcohol consumption typically occurs in either binge (4+/5+ drinks in 2 h) or even higher intensity (8+/10+) drinking episodes which result in high blood ethanol concentrations (BEC) [20]. In order to model human adolescent drinking in both exposure pattern and high BEC, a widely accepted rodent model of adolescent intermittent ethanol (AIE) exposure adopted by the Neurobiology of Adolescent Drinking in Adulthood (NADIA)-NIH funded consortium was used. This AIE model was used in both studies, as described previously [21]. In brief, starting at P25, rats received a single daily intragastric (i.g.) intubation of ethanol (5.0 g/kg) or a comparable volume of water on a 2-day on/2-day off schedule until P54. This exposure window encompasses the adolescent development period in rodents, including both before and after puberty. As animals received no ethanol exposure in adulthood, this model allows for the assessment of long-term effects of adolescent ethanol exposure on the adult brain. Rats were weighed on the first of every 2 consecutive days of gavage treatment to ensure accurate dosing as adolescence is a period of rapid body growth and weight gain. All rats gained weight across adolescence, but neither AIE nor galantamine impacted bodyweight in either the prevention study (study 1, *p* = 0.3 and 0.2, respectively) or restoration study (study 2, *p* = 0.07, 0.8, respectively) [for more details see [19]].

Blood ethanol content (BEC) was assessed from tail bleeds at P38 and P54 using a GM7 Analyzer (Analox; London, UK), as described previously [4]. BECs in both the prevention and restoration studies were comparable to those in previous studies and were not differentially impacted by galantamine treatment (Student’s *t* tests, *p*’s > 0.05; Table 2).

**Table 2:** BECs from prevention (study 1) and restoration (study 2) cohorts^a^

Study	Postnatal day	AIE-Veh	AIE-Gal
**Prevention (study 1)**	P38	172 ± 27 mg/dL	158 ± 25 mg/dL
	P54	124 ± 23 mg/dL	150 ± 13 mg/dL
**Restoration (study 2)**	P38	142 ± 22 mg/dL	171 ± 18 mg/dL
	P54	188 ± 25 mg/dL	195 ± 40 mg/dL

^a^These BECs are also published in Crews et al. [19], which assessed forebrain brain regions

### Galantamine treatment and euthanasia

In both studies, galantamine (4 mg/kg, Sigma Aldrich, #1287755) was administered by intraperitoneal injection (i.p.). In the prevention study (study 1), galantamine or saline (vehicle) was administered 30 min prior to either ethanol or water gavage on every gavage treatment day of the AIE paradigm. In contrast, in the restoration study (study 2), galantamine or vehicle was administered every day consecutively for 2 weeks starting on P57, after the end of the ethanol/water gavage.

Rats were euthanized at P70 (prevention, study 1) or P73 (restoration, study 2) with sodium pentobarbital and perfused with 4% paraformaldehyde/0.1 M PBS. Following, their brains were harvested, sunk in 30% sucrose/0.1 M PBS, and frozen for subsequent histological analyses.

### Immunohistochemistry (IHC)

Brains were serially sectioned as coronal slices on a sliding microtome (MICROM HM450; Thermo Scientific, Austin, TX) with 40 μM thickness. Dorsal hippocampal sections (plates 57-65, [22]) were selected approximately every twelfth section, ensuring a 480 μm inter-section interval with representative sections spanning the septotemporal axis of the dorsal hippocampus, and then stained as free-floating sections. Sections were washed 3 × 5 min in 0.1 M PBS in between each incubation step. All sections were incubated for 30 min in 0.6% hydrogen peroxide/0.1 M PBS to block endogenous peroxidase activity. Afterwards, sections underwent a 1-h (70 °C) incubation with Citra buffer (BioGenex, #HK080-9K) to facilitate antigen retrieval when necessary, and then sections were blocked in 4% normal serum for 30 min, followed by a 48-h incubation at 4 °C in one of the appropriate primary antibodies (Table 3). Sections were incubated in 1:200 biotinylated anti-goat (Vector Laboratories, #BA-5000), anti-rabbit (BA-1000), or anti-mouse (BA-9200) secondary antibodies for 60 min at room temperature, followed by another 60-min incubation in Vectastain® Elite ABC kit (#PK-6100). Labeling was visualized with nickel/cobalt-enhanced diaminobenzidine (DAB), activated by hydrogen peroxide. Negative controls were performed where tissue was developed with secondary and tertiary antibodies, but the primary antibody was omitted (Supplemental Figure 1).

**Table 3:** Antibody information for IHC, IF, and WB

Antibody	Dilution	Vendor	Catalog number	Application
Goat anti-doublecortin	1:400	Santa Cruz Biotech.	sc-8066	IHC
Mouse anti-doublecortin	1:100	Santa Cruz Biotech	sc-271390	IF
Rabbit anti-Ki67	1:200	Abcam	ab66155	IHC
Rabbit anti-cleaved caspase-3	1:10,000 1:5000	Cell Signaling	9661S	IHC IF
Rabbit anti-HMGB1	1: 1000	Abcam	ab18256	IHC
Rabbit anti-COX-2	1:2500	Cell Signaling	12282S	IHC
Mouse anti-CCL2	1:1000	Millipore	MABN712	IHC
Mouse anti-PCNA	1:4000	Cell Signaling	22586	IHC
Rabbit anti-BDNF	1:500	Abcam	ab108319	WB
Rabbit ant-Insulin R β	1:1000	Cell Signaling	23413S	WB
Mouse anti-gp91-phox	1:200	Santa Cruz	sc-130543	WB
Rabbit anti-Pan-Trk	1:1000	Cell Signaling	92991S	WB
Mouse anti-IGF2	1:100	Santa Cruz	sc-515805	WB

*IHC* Immunohistochemistry, *IF* immunofluorescence, *WB* western blot

### Co-label immunofluorescence (IF)

Sections were washed 3 × 5 min in 0.1 M PBS in between each incubation step. All sections underwent a 1-h incubation with Citra buffer (70 °C) to facilitate antigen retrieval when necessary, and then sections were blocked in 4% normal serum for 30 min, followed by a 72-h incubation at 4 °C in mouse anti-doublecortin (1:100) and rabbit anti-cleaved caspase-3 (1:5000) (Table 3). Following, sections were washed and then incubated in goat anti-mouse Alexa Fluor® 488 (Invitrogen, #A-11001; 1:1000) and goat anti-rabbit Alexa Fluor® 594 (Invitrogen, #A-11012; 1:1000).

### Immunohistological quantification

For quantification of dorsal hippocampal immunostaining, photomicrographs of four representative hippocampal sections from each animal were captured using an Olympus BX50 microscope with a 20× objective and a Sony DCX-390 camera and then quantified using BioQuant Nova Advanced Image Analysis software (R&M Biometric, Nashville, TN). Due to multiple endpoints measured in the dorsal hippocampus in the current study and limits on the number of sections, these four sections were analyzed using a modified stereological technique validated for hippocampal counts as described previously [9]. For all immunofluorescence quantification, all images were captured using a Nikon DS-RiZ immunofluorescent microscope (Nikon, Inc., Melville, NY) with a 20× objective and then quantified using the Nikon NIS-Elements AR46 software. All sections were quantified by an experimenter who was blind to all conditions using a modified version of unbiased stereological quantification, which has been extensively validated and published previously [9, 15]. In brief, for all measures, area was defined as the subgranular zone and granular cell layer of both the inferior and superior blades of the dentate gyrus visualized on coronal sections selected using anterior-posterior coordinates from Bregma −3.14 mm to −4.16 mm, with 4 representative sections and a minimum of 12 (IHC) or 32 (IF) pictographs quantified per animal per stain. As previously noted, sections were selected from every twelfth well collected during slicing, ensuring that each section was both random and representative across the septotemporal axis of the dorsal hippocampus with an approximate 480 μm inter-section interval. This analysis allows systematic random sampling across the dorsal hippocampus. For each image, the plane of focus was determined as the center point in the z-plane with a 10-μm dissector which includes the largest number of cells in focus. Cells which would typically fall past the guard zones in traditional unbiased stereology are therefore not in focus and not included in any analyses. This type of random sampling avoids bias due to double-counts and edge effects [9]. DCX was quantified as the sum of the number of positive immunoreactive (+IR) pixels per total area. All other immunoreactive markers were quantified as the sum of IR+ cell numbers per total area. As we have previously reported, these modified parameters produce highly reproducible results which are nearly identical to those used in traditional unbiased stereology [9].

### Immunoblot analysis

Rat hippocampi were rapidly dissected on ice and frozen in liquid nitrogen. Samples were later sonicated in RIPA lysis buffer with a protease inhibitor (PI) cocktail, after which lysates were centrifuged at 21,000 × *g* for 15 min, and then the supernatant was stored at −20 °C. Protein concentrations were determined using a Pierce BCA protein assay. For each gel, protein was diluted with RIPA/PI and loading buffer, and then denatured by boiling for 5 min. For every immunoblot, 40 μg of protein was loaded into every lane of mini-PROTEAN TGX stain-free gels (Bio-Rad Laboratories, Hercules, CA, USA) and then run at 130 V. After which, protein was transferred onto nitrocellulose membranes using Trans-Blot Turbo (Bio-Rad Laboratories, Hercules, CA, USA). Each membrane was imaged using a Revert^TM^ 700 total protein stain (LI-COR Biosciences, Lincoln, NE, USA) to assess accurate transfer and total transferred protein content per sample. Membranes were then washed 3 × 10 min in TBS and blocked in Intercept® blocking buffer (LI-COR Biosciences, #927-60001), after which they were incubated overnight at 4 °C in primary antibody/Intercept® T20 antibody diluent (LI-COR, #927-65001; see Table 3 for concentrations). After 3 × 10-min washes in TBS/0.1% Tween-20, membranes were incubated in an appropriate LI-COR IRDye® secondary antibody for 1.5 h. Membranes were washed and imaged using the LI-COR Odyssey® imaging system. Total fluorescence for each sample was quantified at the appropriate molecular weights and then normalized relative to transferred total protein content for that sample, as assessed by Revert^TM^ 700 using the LI-COR Odyssey imaging system. For pictographs of each immunoblot, see Supplemental Figure 2.

### Statistical analyses

All statistical analyses were assessed using 2 × 2 analysis of variance with the SPSS® software. Post hoc follow-up analyses were conducted when appropriate using a Bonferroni correction. Follow-up comparisons for ethanol effects which have been reported previously were assessed as determined *a priori*. For all statistical measures, *α* = 0.05. All results are expressed as the mean ± standard error of the mean (SEM). Outliers were assessed using Grubbs outlier analyses tests and removed when applicable.

## Results

### Study 1 (prevention): Galantamine prevents AIE-induced deficits in hippocampal neurogenesis

**Fig. 2:**
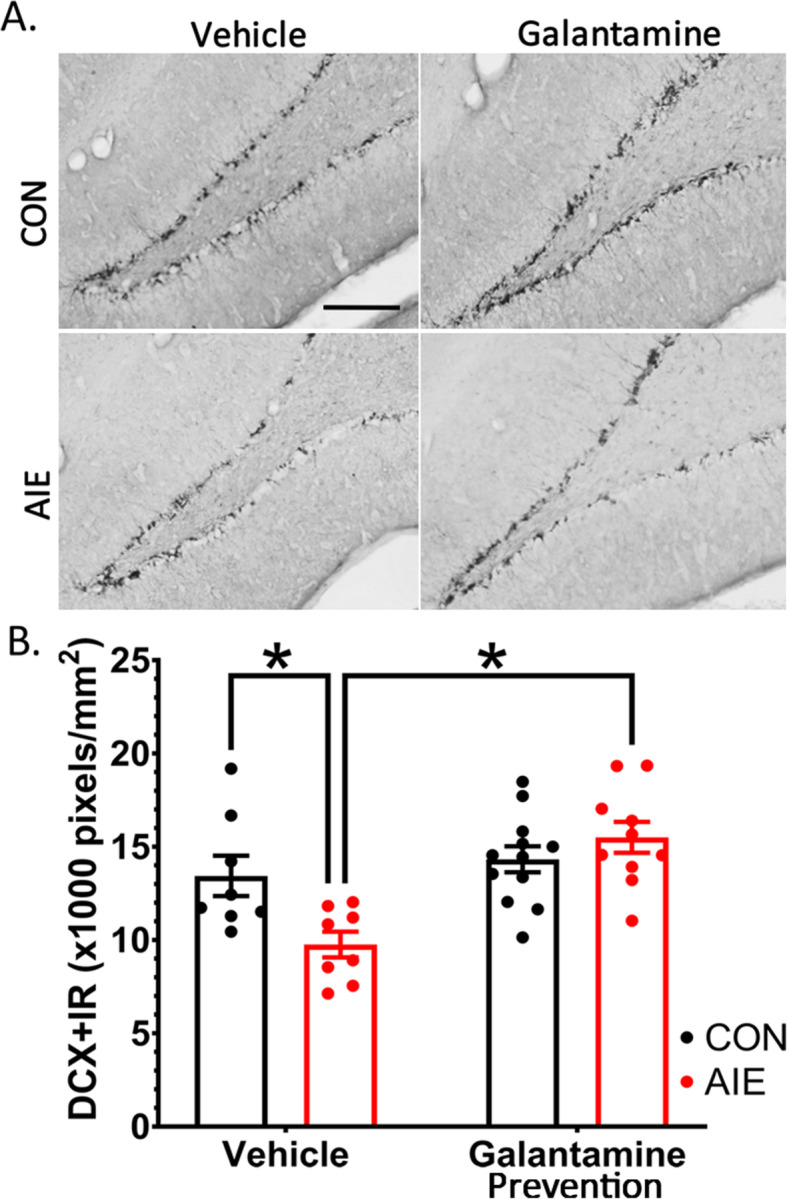
Galantamine prevents AIE-induced deficits in hippocampal neurogenesis. (**A**) Photomicrograph of DCX+IR in each respective group. Clockwise, from the upper left corner: CON-Veh, CON-Gal, AIE-Gal, AIE-Veh. (**B**) AIE decreased DCX+IR relative to CON rats in vehicle-treated groups. Galantamine restored DCX+IR to control levels in AIE-treated rats. This suggests that cholinesterase inhibition blocks the long-term effects of AIE on neurogenesis within the dentate gyrus. DCX+IR was quantified as pixels/mm^2^ with results expressed as mean ± standard error of the mean (SEM). Scale bars indicate 200 μM. For each identified comparison, *p < 0.05

Doublecortin (DCX) is a microtubule-associated protein unique to early neuronal progenitor cells. In previous studies, density of DCX immunoreactivity (IR) was expressed in neuroprogenitor cells that incorporated bromodeoxyuridine (BrdU) into their DNA during mitosis, and acute ethanol exposure-induced loss of DCX+IR which paralleled loss of BrdU+IR [8]. In the current study, we tested the hypothesis that daily galantamine pretreatment before ethanol exposure would prevent AIE-induced reductions of adult DCX+IR neurons in the dentate gyrus. Analysis of variance indicated that galantamine pretreatment interacted with ethanol exposure to impact DCX+IR in the dentate gyrus, *F*(1, 34) = 8.45, *p* = 0.006. Post hoc analyses indicated that AIE reduced DCX+IR by 27% relative to controls in vehicle-treated rats, *p* = 0.006. Conversely, galantamine significantly increased DCX+IR by 43% within AIE-exposed rats, *p* < 0.001 (Fig. 2, Table 4). These results indicate that galantamine pretreatment can prevent AIE-induced deficits in hippocampal neurogenesis.

**Table 4:** Study 1: Percent change in DCX+IR relative to CON-Veh rats

Neurogenesis marker	CON-Veh	CON-Gal	AIE-Veh	AIE-Gal
DCX	100.0%	106.6%	**72.6%**^**a**^	**115.4%**^**b**^

Immunohistochemical stain (pixels/mm^2^). Data are expressed as the % change relative to CON-Veh cohorts

^a^Significantly different from CON-Veh

^b^Significantly different from AIE-Veh

### Study 1 (prevention): mechanisms of AIE-reduced adult neurogenesis and galantamine prevention

**Fig. 3:**
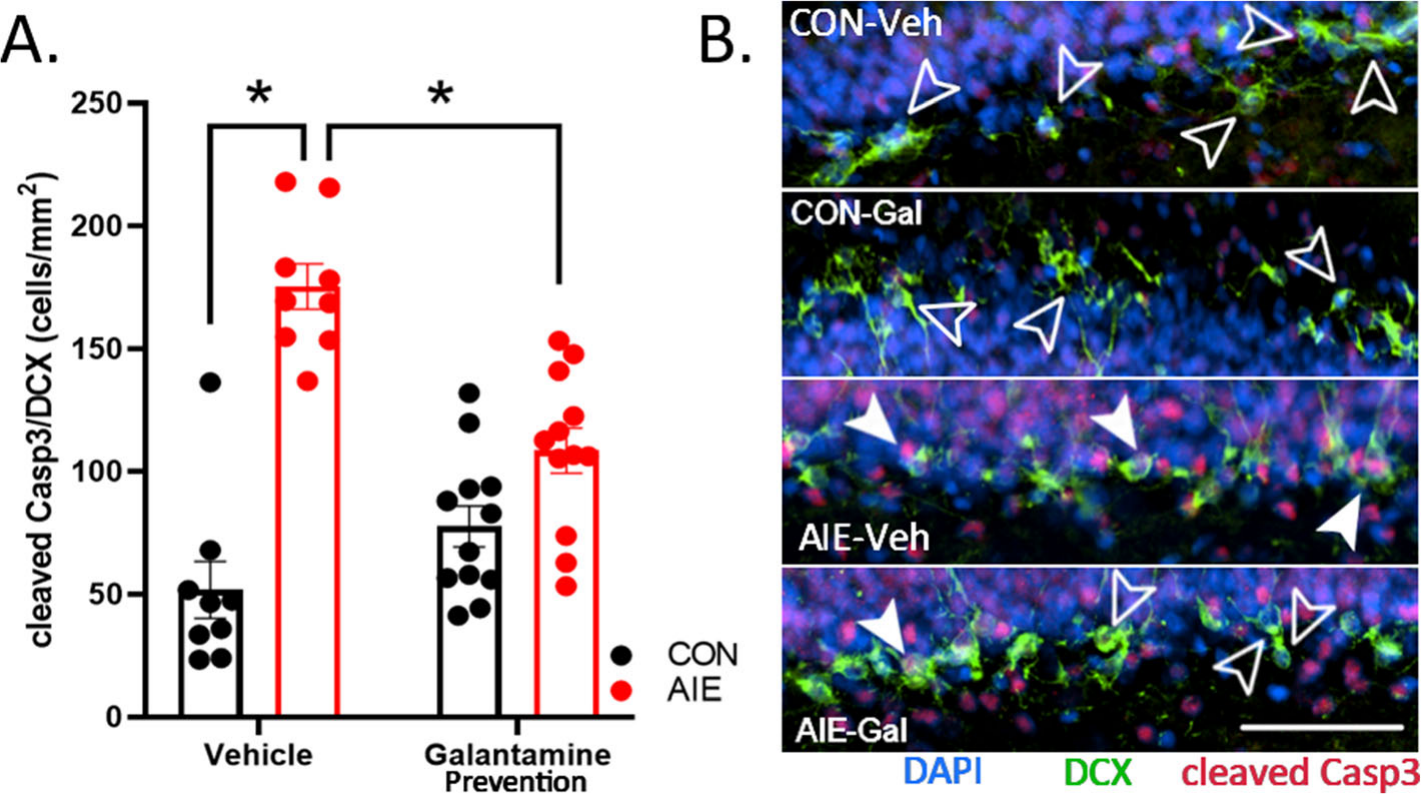
AIE increases in cleaved Casp3 in DCX-expressing immature neurons are prevented by galantamine. (**A**) Cleaved Casp3 is a marker of activated cell death machinery, and DCX is a marker of immature neurons. We analyzed co-labeling of cleaved Casp3 in DCX cells as an indicator of cell death in these immature neurons. AIE significantly increased cleaved Casp3+IR in vehicle-exposed rats. Excitingly, galantamine reversed this effect, indicating that galantamine pretreatment prevents AIE induction of cell death cascades in immature neurons. (**B**) Photomicrographs include cleaved Casp3+IR (red) with DCX (green) and DAPI (blue). Closed white arrows indicate cleaved Casp3 in DCX cells. Open arrows outlined in white indicate DCX cells without cleaved Casp3. Scale bars indicate 100 μM. For each identified comparison, *p < 0.05

Overarching shifts in neurogenesis require either changes within neuroprogenitor proliferation or activation of cell death pathways in maturing neurons. To investigate the mechanisms of AIE-induced loss of DCX+IR and interaction with galantamine, we assessed progenitor division (Ki67+IR), which is a marker for cells in the active phase of the cell cycle (M, G_2_, S, and G1) [23], and we assessed cleaved caspase-3+IR (Casp3), a key executioner of cell death cascades, when selectively expressed within DCX-expressing immature granule cells. There was an interaction between AIE and galantamine treatment on cleaved Casp3+ cells co-localized with DCX, *F*(1, 38) = 23.33, *p* < 0.001. Specifically, cleaved-Casp3 was significantly increased in DCX-labeled cells in AIE-Veh rats compared to CON-Veh rats, *p* < 0.001. Excitingly, galantamine reversed this effect, significantly decreasing cleaved Casp3 co-localization in AIE cohorts, *p* = 0.019. Thus, one mechanism by which galantamine rescues neurogenesis is by reducing Casp3 activation in maturing newborn neurons (Fig. 3, Table 5).

**Table 5:** Study 1: Percent change in cleaved Casp3 colocalization with DCX relative to CON-Veh rats

Marker	CON-Veh	CON-Gal	AIE-Veh	AIE-Gal
Cleaved Casp3/DCX	100.0%	149.8%	**337.76%**^**a**^	**209.0%**^**b**^

Immunohistochemical stains (cells/mm^2^). Data are expressed as the % change relative to CON-Veh cohorts

^a^Significantly different from CON-Veh

^b^Significantly different from AIE-Veh

**Fig. 4:**
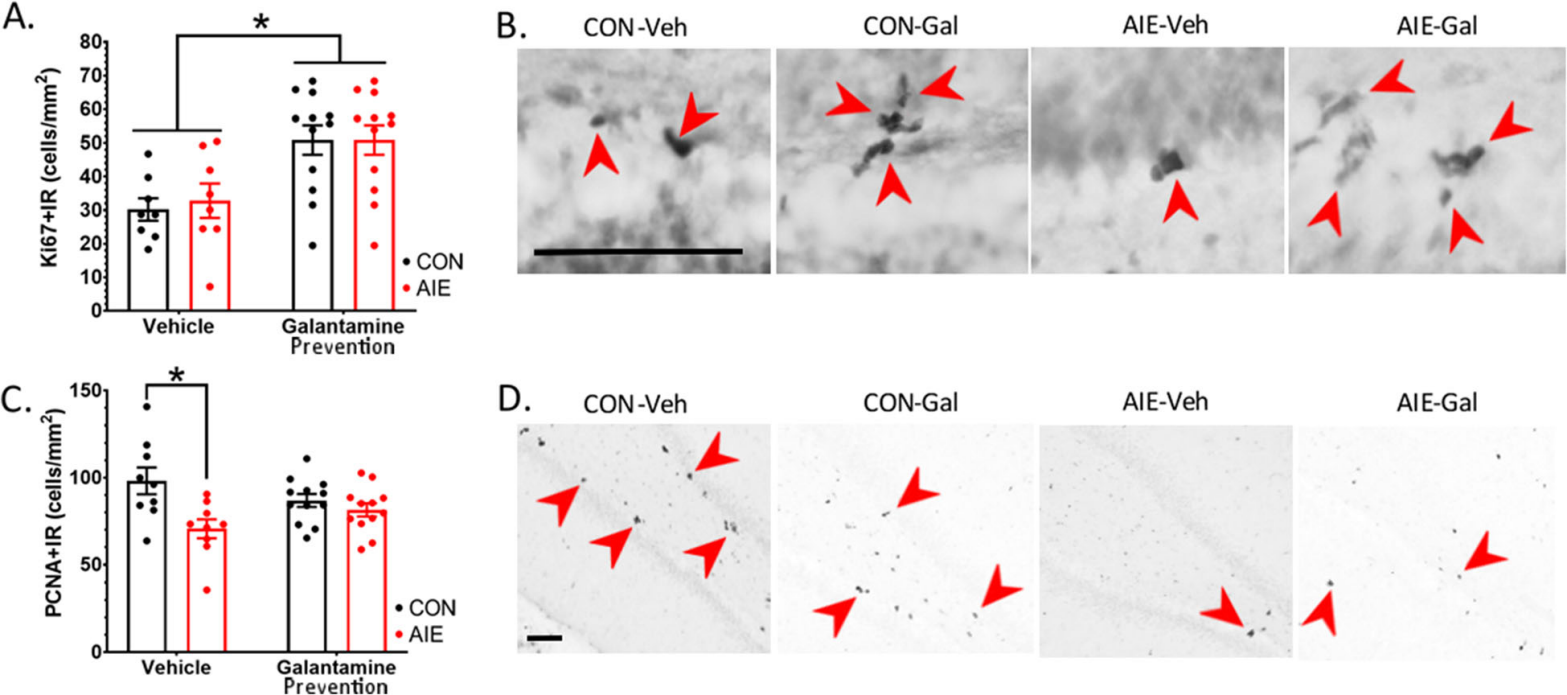
Galantamine and AIE differentially impact expression of Ki67 and PCNA. (**A**) Ki67 is a marker of cell proliferation as it is expressed during the active phases of the cell cycle. Results indicate that galantamine significantly increased Ki67+IR, suggesting that galantamine increases cell proliferation. (**B**) Photomicrographs of Ki67. Ki67 is expressed in the subgranular zone of the dentate with cells appearing as small clusters. (**C**) PCNA is highly expressed in cells undergoing both cell proliferation processes as well as cell repair. AIE decreased PCNA+IR relative to controls, and this effect was not reversed by galantamine. (**D**) Photomicrographs of PNCA+IR. PCNA is highly expressed in the subgranular zone of the dentate. Scale bars indicate 100 μM. Red arrows indicate either Ki67+IR cells (**B**) or PCNA+IR cells (**D**). All results are expressed as mean ± SEM. For each identified comparison, *p < 0.05

When we assessed cell proliferation using the marker Ki67+IR, there was no effect of AIE, *p* > 0.05. In contrast, galantamine pretreatment significantly increased Ki67+IR in both AIE and CON groups relative to vehicle-treated rats, *F*(1, 38) = 14.09, *p* = 0.001. This suggests that galantamine increases Ki67+IR cell proliferation regardless of ethanol exposure. Interestingly, galantamine did not impact the overarching number of clusters of proliferating cells, *F*(1, 38) = 4.08, *p* > 0.05, suggesting galantamine may be shifting the duration of time cells spend in the active phase of cell division to yield greater cell division rather than increasing the number of cells which are active progenitors (Fig. 4A, B; Table 6).

**Table 6:** Study 1: Percent change in cell cycle markers relative to CON-Veh rats

Cell cycle marker	CON-Veh	CON-Gal	AIE-Veh	AIE-Gal
Ki67	100.0%	**173.1%**^**a**^	120.1%	**163.2%**^**a**^
PCNA	100.0%	88.6%	**72.0%**^**a**^	83.1%

Immunohistochemical stains (cells/mm^2^). Data are expressed as the % change relative to CON-Veh cohorts

^a^Significantly different from CON-Veh

^b^Significantly different from AIE-Veh

To further elucidate the effects of AIE and galantamine on the cell cycle, we examined proliferating cell nuclear antigen (PCNA), which marks DNA replication [24] as well as DNA repair, thus identifying many more cells than Ki67+IR. Results indicated that there was a significant interaction between adolescent exposure to ethanol/water and pretreatment with galantamine/vehicle on PCNA+IR in the hippocampus, *F*(1, 38) = 4.72, *p* = 0.04. Bonferroni-corrected post hoc analyses indicate that this effect was primarily driven within the vehicle-treated groups where AIE-Veh rats exhibited significantly decreased PCNA+IR relative to CON-Veh rats, *p* = 0.001 (Fig. 4C, D; Table 6). The distinction in results between PCNA and Ki67 markers suggests differences in labeling of cell populations, perhaps distinguishing between proliferating and/or DNA-repairing cell mechanisms. In sum, results suggest that AIE impairs neurogenesis through increases in activation of cell death pathways, and galantamine rescues AIE reductions in neurogenesis by driving cell proliferation.

### Study 1 (prevention): AIE increases in hippocampal CCL2+IR are prevented by galantamine

**Fig. 5:**
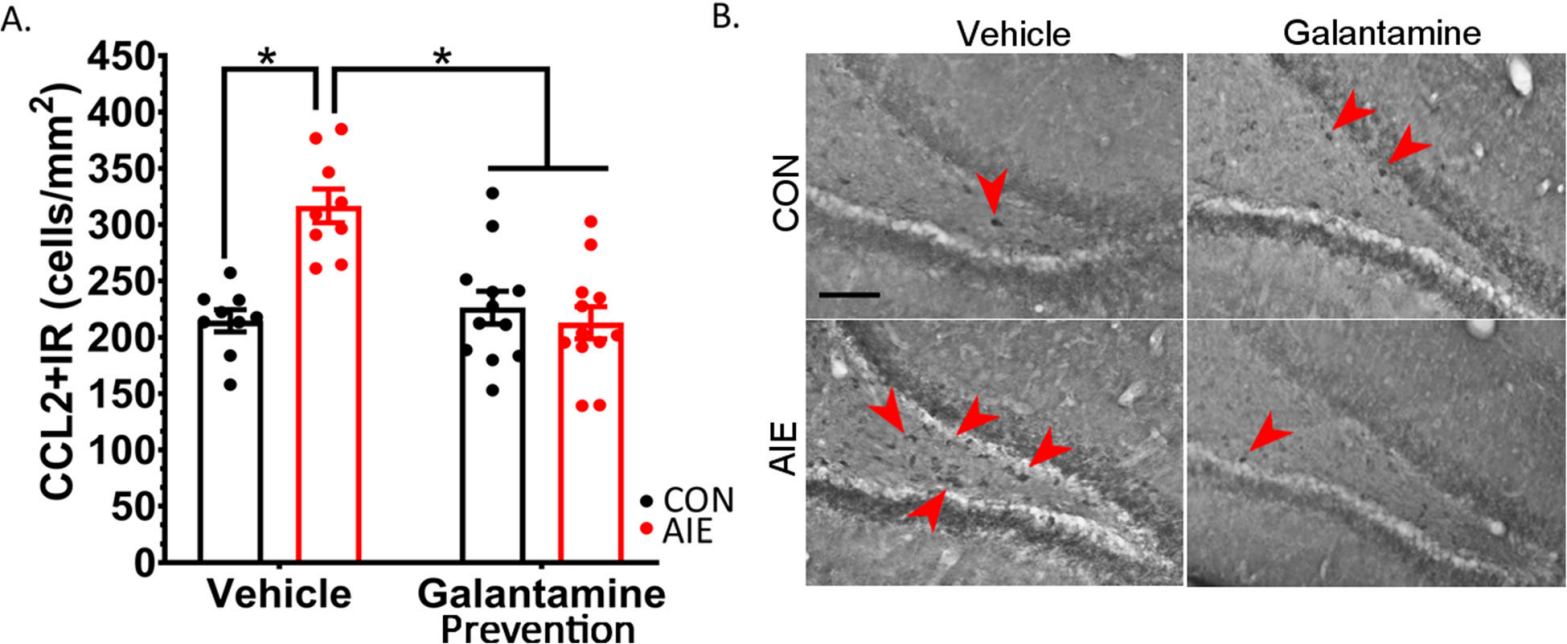
Galantamine blocks AIE induction of chemokine (C-C motif) ligand 2 (CCL2). CCL2 is a chemokine that can also function as a neuromodulator to impact neuronal excitability and impair neurogenesis. (**A**) The current study found that galantamine blocked AIE induction of CCL2 in the hilus, suggesting that increasing acetylcholine signaling combats ethanol-induced proinflammatory cascades. (**B**) Example photomicrographs taken at 20× magnification of each group: CON-Veh, CON-Gal, AIE-Veh, AIE-Gal. There are more CCL2+IR cells in AIE-treated rats. Results are expressed as mean ± SEM. Scale bars indicate 100 μM. Red arrows indicate CCL2+IR cells. For each identified comparison, *p < 0.05

We hypothesized that the galantamine-increased cholinergic anti-inflammatory signaling in the hippocampus would reduce AIE induction of pro-inflammatory signaling cascades and the consequential loss of hippocampal neurogenesis. Monocyte chemoattractant protein-1/chemokine (C-C motif) ligand 2 (CCL2) is increased in post-mortem human AUD brain [25] and is implicated in the regulation of neurogenesis [26]. CCL2 is a chemokine, and its receptor (CCR2) is robustly expressed on the surface of progenitor cells within the subgranular zone of the dentate gyrus [27]. Excitingly, we report here for the first time that AIE increases CCL2+IR by 48% more than controls in the adult hippocampus, *p* < 0.001. Furthermore, galantamine interacted with AIE to prevent ethanol’s induction of CCL2, *F*(1, 38) = 16.91, *p* < 0.001. There was no difference between AIE and control rats with galantamine pretreatment, *p* = 0.47. Co-immunofluorescence labeling further indicates that the majority of hilar CCL2 staining is neuronal (Supplemental Figure 3); thus, as the cell counts performed here included only large cells, this quantitation likely reflects neuronal rather than glial induction of CCL2+IR. This suggests that AIE increases large DG hilar CCL2+IR cells that could contribute to AIE-reduced DCX+IR, potentially through CCL2-CCR2 signaling. In sum, AIE increases in proinflammatory CCL2+IR were prevented by galantamine, which is consistent with overarching findings that restoration of AIE’s disruption of the proinflammatory-trophic balance is necessary for normal hippocampal neurogenesis (Fig. 5, Table 7).

**Table 7:** Study 1: Galantamine prevention of hippocampal neuroimmune induction after AIE

Neuroimmune	CON-Veh	CON-Gal	AIE-Veh	AIE-Gal
CCL2	216 ± 14 (100%)	226 ± 12 (105%)	**316 ± 14 (148%)**^**a**^	**212 ± 12 (99%)**^**b**^
HMGB1	780 ± 97 (100%)	820 ± 88 (103%)	**1092 ± 102 (137%)**^**a**^	**761 ± 88 (95%)**^**b**^
COX-2	289 ± 17 (100%)	280 ± 16 (98%)	**349 ± 18 (119%)**^**a**^	315 ± 15 (109%)

Immunohistochemical stains (cells/mm^2^). Data is expressed as mean ± standard error of the mean (SEM); % change relative to CON-Veh rats are represented in parentheses to the right of each datapoint

^a^Significantly different from CON-Veh

^b^Significantly different from AIE-Veh

To further explore the effects of AIE and galantamine on proinflammatory markers, we also assessed high mobility group box protein 1 (HMGB1) and cyclooxygenase-2 (COX-2). HMGB1 is a histone-binding protein which initiates proinflammatory cascades. Results indicate that there was a trend for AIE and galantamine to interact on HMGB1+IR, *F*(1, 38) = 3.89, *p* = 0.06. As HMGB1 has been shown to be significantly increased after AIE in multiple studies [12, 15], we compared CON-Veh to AIE-Veh as determined *a priori*. AIE significantly increased HMGB1+IR by 37% over that of controls in vehicle-treated cohorts, *p* = 0.04. Galantamine blocked this effect, significantly reducing HMGB1+IR within AIE-treated rats to control levels, *p* = 0.01 (Table 7).

Similarly, COX-2 is an inducible enzyme which is upregulated in glutamatergic neurons in response to NF-ĸB induction of proinflammatory cascades as well as locally in response to increased intracellular calcium levels following NMDA receptor activation. AIE significantly increased COX-2+IR in the dentate, *F*(1, 38) = 7.91, *p* = 0.01. More specifically, AIE-Veh rats exhibited a 19% increase in levels of COX-2+IR relative to water-treated controls, *p* = 0.02. AIE rats exposed to galantamine were not significantly different from any group, indicating that galantamine partially reversed this effect, *p* > 0.05. For collective results on the effects of AIE and galantamine on these markers, see Table 7. In sum, AIE induced the proinflammatory markers CCL2+IR, HMGB1+IR, and COX2+IR and reduced DCX+IR, which are consistent with the hypothesis that increases in neuroimmune signaling contribute to loss of neurogenesis. Galantamine treatment in conjunction with AIE reversed the induction of these proinflammatory genes and prevented loss of DCX+IR, further suggesting that cholinergic signaling is an important mediator in the loss and restoration of both the proinflammatory environmental milieu in the neurogenic niche and also neurogenesis after AIE.

### Study 2 (restoration): AIE followed by galantamine restores hippocampal neurogenesis

**Fig. 6:**
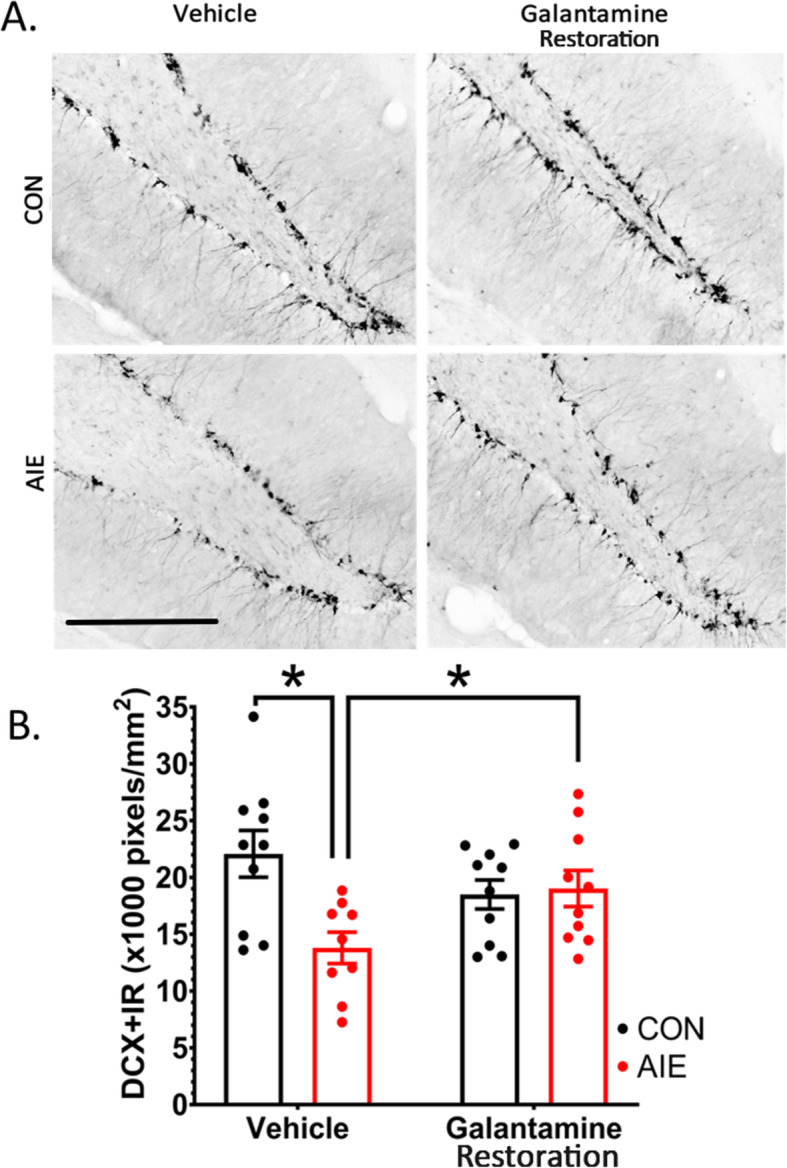
Galantamine reverses AIE-induced deficits in hippocampal neurogenesis. (**A**) Photomicrograph of DCX+IR in each respective group. Clockwise, from the upper left corner: CON-Veh, CON-Gal, AIE-Gal, AIE-Veh. (**B**) AIE decreased DCX+IR relative to CON rats in vehicle-treated groups. Galantamine restored DCX+IR to control levels in AIE-treated rats. This indicates that cholinesterase inhibition reverses the long-term effects of AIE on neurogenesis within the dentate gyrus. DCX+IR was quantified as pixels/mm^2^ with results expressed as mean ± standard error of the mean (SEM). Scale bars indicate 200 μM. For each identified comparison, *p < 0.05

To determine if galantamine treatment after the end of the AIE paradigm can restore AIE-induced deficits in hippocampal neurogenesis, we initiated galantamine treatment 3 days after AIE ended. As in the prevention study (study 1), we used DCX+IR as an indicator of neurogenesis. Excitingly, results indicated that there was an interaction between AIE and galantamine post-treatment on DCX+IR, *F*(1, 35) = 8.90, *p* = 0.005. Post hoc analyses revealed that within vehicle-treated groups, AIE decreased DCX+IR by 37% relative to water-treated controls, *p* = 0.001. Galantamine was able to rescue AIE-induced deficits, significantly increasing DCX+IR by 26% within AIE-treated rats, *p* = 0.02, resulting in no significant differences between CON-Veh and CON-Gal-treated rats. This indicates that galantamine reverses AIE reductions in adult hippocampal neurogenesis (Fig. 6, Table 8).

**Table 8:** Study 2: Percent change in DCX+IR relative to CON-Veh rats

Neurogenesis marker	CON-Veh	CON-Gal	AIE-Veh	AIE-Gal
DCX	100.0%	82.5%	**62.2%**^**a**^	**88.2%**^**b**^

Immunohistochemical stains (pixels/mm^2^). Data are expressed as the % change relative to CON-Veh cohorts

^a^Significantly different from CON-Veh

^b^Significantly different from AIE-Veh

### Study 2 (restoration): mechanisms of AIE induction of cell death cascades and reduction in neurogenesis

**Fig. 7:**
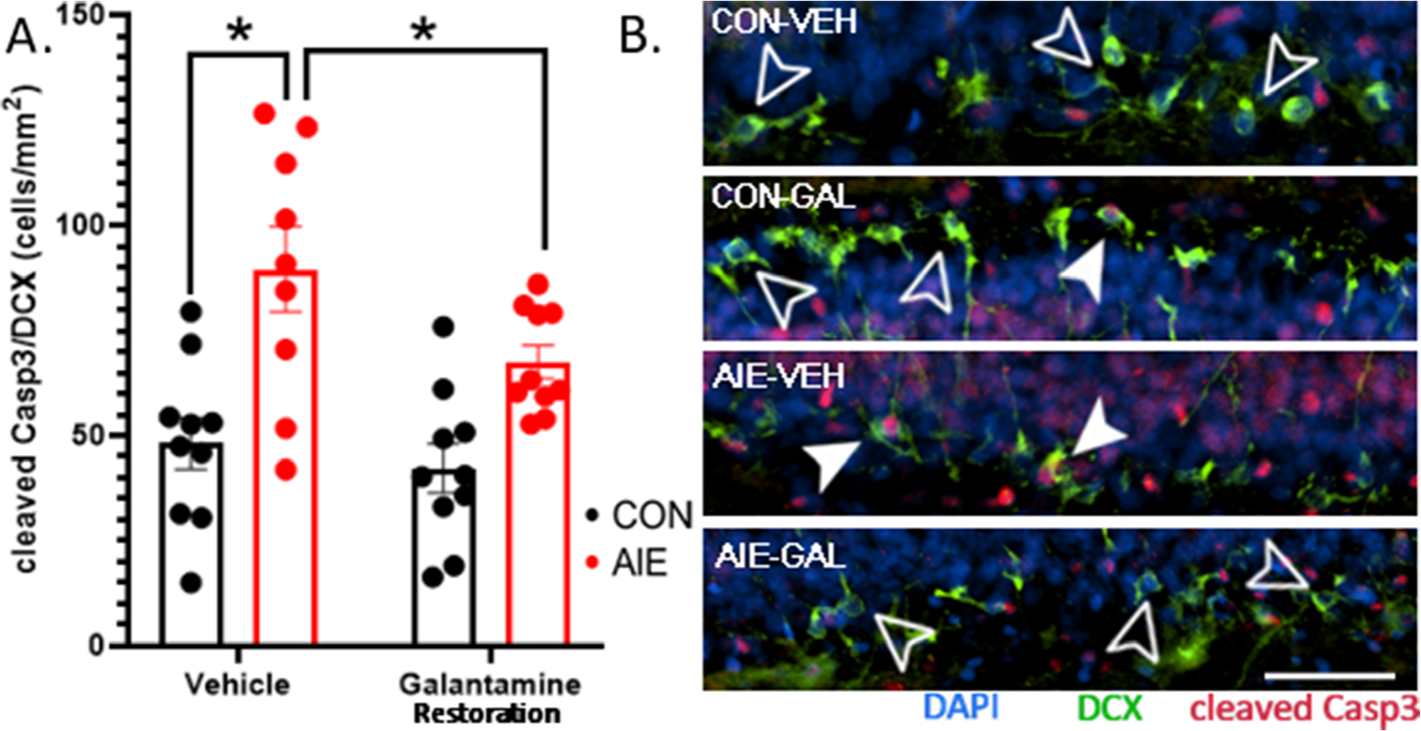
AIE induction of cell death cascades in DCX-expressing neurons are reversed by galantamine. (**A**) AIE increased the number of cells expressing the activated form of Casp3 within DCX-labeled cells, indicating increased apoptosis in immature neurons. As with the prevention study, galantamine reversed this effect. (**B**) Photomicrographs include cleaved Casp3+IR (red) with DCX (green) and DAPI (blue). Closed white arrows indicate cleaved Casp3 in DCX cells. Open arrows outlined in white indicate DCX cells without cleaved Casp3. Scale bars indicate 100 μM. For each identified comparison, *p < 0.05

To gain insight into the mechanisms of DCX+IR restoration by galantamine, we used co-label immunofluorescence to assess cleaved Casp3 expression in DCX, as an indicator of AIE and galantamine’s effects on cell death cascades in immature neurons. AIE significantly increased Casp3 activation in DCX-labeled cells relative to water-treated controls, *F*(1, 35) = 4.43, *p* = 0.043. Follow-up analyses based on *a priori* assessments indicated that as in study 1 (prevention), AIE-Veh rats exhibited significantly increased (186%) cleaved Casp3 co-localization with DCX-expressing cells relative to CON-Veh rats, *p* < 0.001. Similarly, galantamine treatment reduced cleaved Casp3 co-localization with DCX-expressing cells in AIE rats, *p* = 0.019. These results indicate that galantamine can rescue activation of cell death cascades after AIE in immature neurons (Fig. 7A, B).

### Study 2 (restoration): AIE and galantamine impact proliferation markers Ki67 and PCNA+IR

**Fig. 8:**
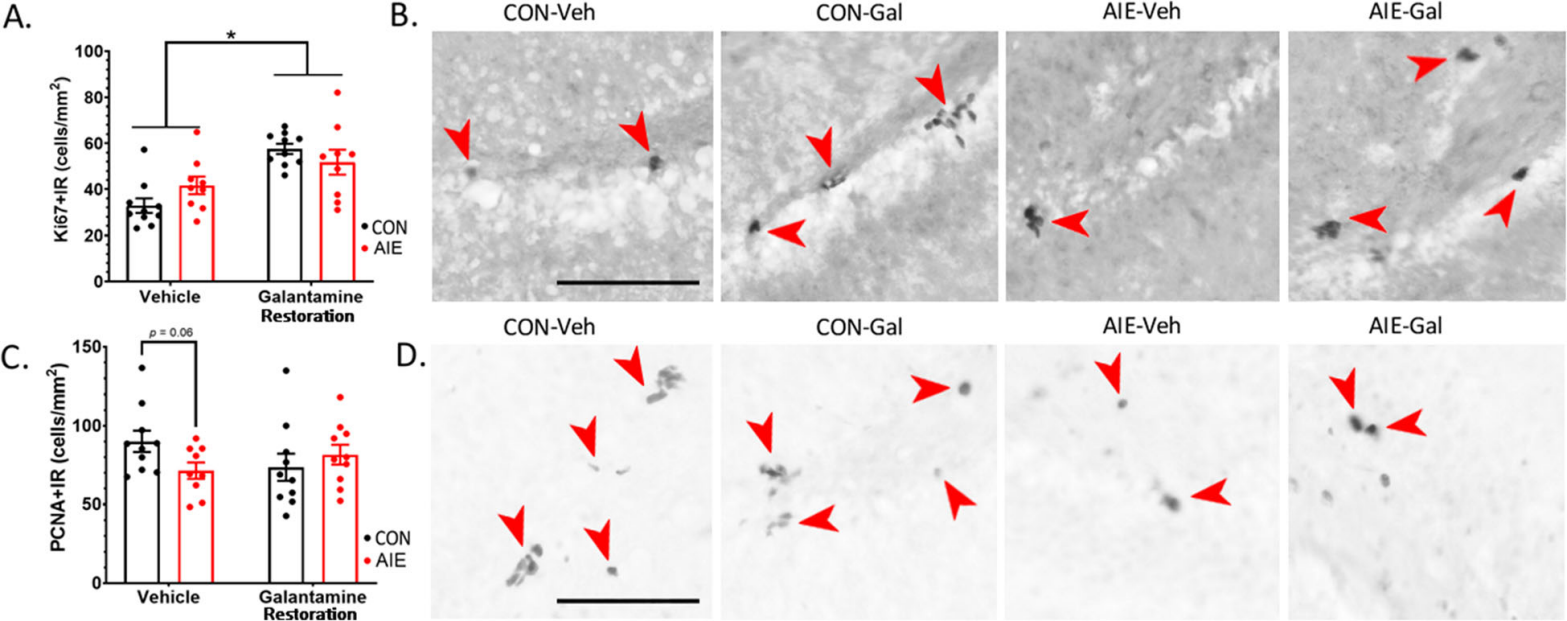
Galantamine increases Ki67 but not PCNA in the subgranular zone of the dentate. (**A**) Ki67 is a fairly specific marker of cell proliferation which is upregulated in the active phase of the cell cycle. Similar to the prevention study, we found that 2 weeks of galantamine treatment increased Ki67+IR in the subgranular zone, independent from adolescent exposure to ethanol or water. (**B**) Representative photomicrographs were taken of dentate Ki67+IR from each group (20× magnification), demonstrating that galantamine treatment increases Ki67+IR, regardless of adolescent exposure condition. (**C**) While PCNA is expressed during cell proliferation, it is also expressed when DNA is undergoing cell repair. Here, we found that AIE decreased PCNA+IR relative to water-treated controls in vehicle-treated cohorts. The divergence of these findings from Ki67 could suggest that AIE is decreasing the induction of appropriate cell repair machinery in response to DNA damage. (**D**) Photomicrographs of PCNA+IR in the dentate gyrus. A representative photomicrograph of the hippocampal dentate gyrus was taken at 20× magnification for each group. Results are expressed as mean ± SEM. Scale bars indicate 100 μM. For each identified comparison, *p < 0.05

To investigate the effects of AIE and galantamine on proliferation markers in the dentate gyrus, Ki67+IR and PCNA+IR were quantified. There was a main effect of galantamine on Ki67+IR, *F*(1, 34) = 21.51, *p* < 0.001. Specifically, galantamine increased Ki67+IR regardless of prior adolescent exposure to ethanol or water. Conversely, AIE followed by galantamine showed a trend toward reduced PCNA+IR, *F*(1, 35) = 3.71, *p* = 0.06. Based on *a priori* assessment of AIE effects independent of galantamine, an independent samples *t* test indicated that PCNA+IR was significantly decreased in AIE rats compared to CON rats, *t*(17) = 2.14, *p* = 0.04. These results are similar to results from the prevention study (study 1), where amount of Ki67+IR and PCNA+IR suggests unique populations of cells which are differentially sensitive to effects from galantamine and AIE (Fig. 8, Table 9).

**Table 9:** Study 2: Percent change in cell cycle markers relative to CON-Veh rats

	CON-Veh	CON-Gal	AIE-Veh	AIE-Gal
Ki67	100.0%	**175.2%**^**a**^	126.8%	**157.6%**^**a**^
PCNA	100.0%	81.7%	**79.3%**^**a**^	90.6%

Immunohistochemical stains (cells/mm^2^). Data are expressed as the % change relative to CON-Veh cohorts

^a^Significantly different from CON-Veh

^b^Significantly different from AIE-Veh

### Study 2 (restoration): Galantamine increases hippocampal pan-Trk expression in AIE-treated animals

Both insulin-signaling and brain-derived neurotrophic factor (BDNF) have important trophic support roles that can impact neurogenesis. Using western blot analysis, we tested whether AIE and galantamine impacted expression of insulin-like growth factor-2 (IGF2), insulin receptor β (IRβ), pro- and mature BDNF, and pan-Trk receptors, which could impact neurogenesis by shifting trophic signaling. There was no effect of AIE or galantamine on pro-BDNF, *F*(1, 25) = 1.98, *p* = 0.17, or mature BDNF, *F*(1, 25) = 0.76, *p* = 0.39. However, galantamine treatment and AIE exposure did interact with pan-Trk, which includes receptors TrkA, TrB, and TrkC, *F*(1, 25) = 5.78, *p* = 0.02. Specifically, galantamine increased hippocampal pan-Trk expression in AIE-treated rats, *p* = 0.04. This indicates that galantamine may improve trophic signaling in AIE-treated rats. Similarly, there was no impact of either ethanol or galantamine in expression of IGF2, *F*(1, 25) = 0.31, *p* = 0.58, its precursor, *F*(1, 25) = 0.07, *p* = 0.80, or IRβ, *F*(1, 25) = 0.31, *p* = 0.58. This suggests that AIE-induced loss of neurogenesis is not due to loss in hippocampal expression of these markers of trophic support (Table 10).

**Table 10:** Study 2: Differential impact of AIE and galantamine on trophic and proinflammatory factors

Immunoblot marker	CON-Veh	CON-Gal	AIE-Veh	AIE-Gal
Pro-BDNF	100% ± 12	97% ± 12	111% ± 14	123% ± 13
Mature BDNF	100% ± 12	116% ± 12	94% ± 14	87% ± 13
Pan-Trk	100% ± 16	75% ± 15	87% ± 20	**136% ± 9**^**b**^
IRβ	100% ± 11	98% ± 11	90% ± 13	103% ± 12
IGF2 Precursor	100% ± 10	127% ± 30	93% ± 15	101% ± 15
Mature IGF2	100% ± 20	86% ± 20	104% ± 23	79% ± 21
Gp91^phox^	100% ± 17	77% ± 16	**131% ± 19**^**a**^	**120% ± 17**^**a**^

Immunoblot quantification using Li-Cor Odyssey. Data is expressed as mean ± standard error of the mean (SEM) with data expressed as a percentage of CON-Veh

^a^Significantly different from CON-Veh

^b^Significantly different from AIE-Veh, *p* < 0.05

### Study 2 (restoration): Galantamine reverses AIE induction of proinflammatory cascades

**Fig. 9:**
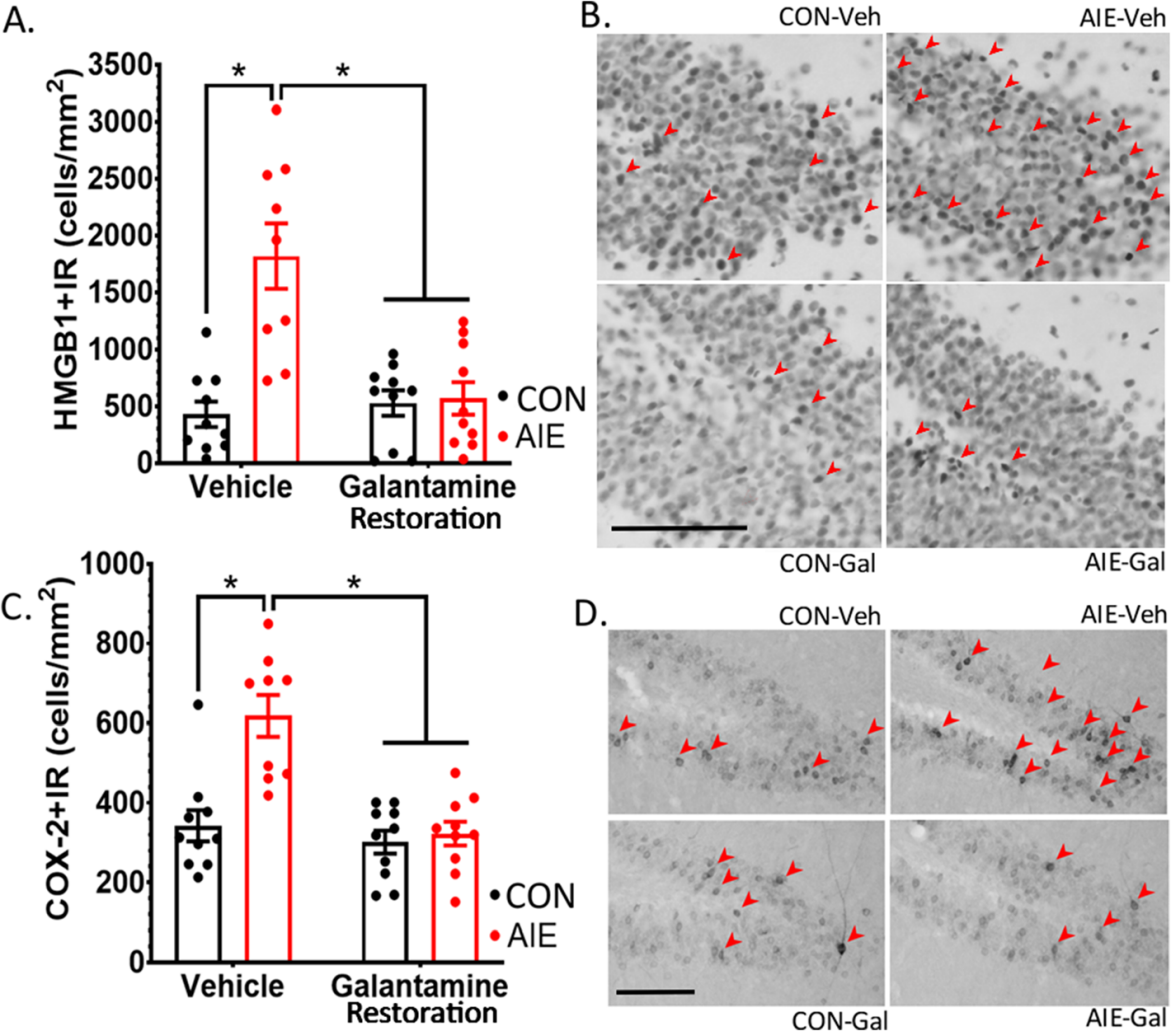
Galantamine reverses AIE induction of proinflammatory cascades. Two proinflammatory markers which have been shown to be upregulated by ethanol acutely are HMGB1 and COX-2. We tested whether galantamine could reverse chronic upregulation of these markers after AIE. (**A**) AIE increases HMGB1+IR, and this effect is reversed by galantamine. (**B**) Photomicrographs of HMB1 immunoreactivity. Clockwise, from the upper left corner: CON-Veh, AIE-Veh, AIE-Gal, CON-Gal. (**C**) AIE increases HMGB1+IR, and this effect is reversed by galantamine. (**D**) Photomicrographs of COX-2 immunoreactivity. Clockwise, from the upper left corner: CON-Veh, AIE-Veh, AIE-Gal, CON-Gal. Results are expressed as mean ± SEM. Red arrows indicate either HMGB1+IR cells (B) or COX-2+IR cells (**D**). Scale bars indicate 100 μM. For each identified comparison, *p < 0.05

Persistent induction of proinflammatory markers is emerging as a hallmark feature in the AIE pathogenesis. Therefore, we tested whether galantamine similarly reverses ethanol’s induction of the proinflammatory factors HMGB1 and COX-2 within the dentate gyrus. In accord with our hypothesis, AIE and galantamine interacted to impact HMGB1+IR in the dentate gyrus, *F*(1, 35) = 10.17, *p* = 0.003. Specifically, AIE increased HMGB1+IR to 424% of that in CON-Veh rats, *p* < 0.001. Galantamine treatment reversed this effect, such that galantamine reduced HMGB1+IR in AIE-treated rats to values similar to CON-Veh rats, *p* < 0.001. There was no effect of galantamine on HMGB1+IR within water-treated controls, *p* = 0.97 (Fig. 9A, B; Table 11). Results from the current study also indicated that AIE and galantamine treatment interacted to impact COX-2+IR, *F*(1, 35) = 11.23, *p* = 0.002. Specifically, AIE increased COX-2+IR to 80% above CON-Veh levels, *p* < 0.001. Galantamine reversed this effect, significantly reducing COX-2+IR within AIE-treated cohorts, *p* < 0.001. Collectively, these results suggest that galantamine reverses induction of proinflammatory cascades within the dentate after AIE (Fig. 9 C, D; Table 11). Thus, AIE causes increases in proinflammatory proteins HMGB1+IR and COX-2+IR that persist into adulthood in association with losses in DCX+IR. These results are consistent with the hypothesis that AIE-induced disruption of the environmental milieu in the neurogenic niche toward a proinflammatory state is an important underlying mechanism of DCX+IR loss and thus a critical mediator of galantamine’s reversal of this loss.

**Table 11:** Study 2: Galantamine reversal of hippocampal neuroimmune induction after AIE

Neuroimmune	CON-Veh	CON-Gal	AIE-Veh	AIE-Gal
HMGB1	531 ± 129 (100%)	538 ± 129 (101%)	**1448 ± 136 (273%)**^**a**^	**601 ± 129 (113%)**^**b**^
COX-2	342 ± 37 (100%)	294 ± 38 (86%)	**602 ± 42 (176%)**^**a**^	**324 ± 38 (95%)**^**b**^

Data are expressed as mean ± standard error of the mean (SEM); % change relative to CON-Veh rats are represented in parentheses to the right of each datapoint

^a^Significantly different from CON-Veh

^b^Significantly different from AIE-Veh

We then used western blots to assess the effects of AIE and galantamine on the catalytic subunit of superoxide-generating enzyme NOX2 (gp91^phox^) as an indicator of a proinflammatory response and oxidative stress typically associated with microglia. Importantly, oxidative stress has been linked to the induction of cell death cascades in various capacities, including microglial phagocytosis [28, 29]. AIE significantly increased gp91^phox^ by 25% over levels in CON-Veh rats, *F*(1, 24) = 4.63, *p* = 0.04. Galantamine did not interact with AIE to reverse this effect, *F*(1, 24) = 0.12, *p* = 0.73 (Table 10). Collectively, results from both the prevention and restoration studies established that AIE reduces DCX+IR likely through increasing cell death cascades, which may be mediated by AIE increases in oxidative damage.

## Discussion

The overarching conclusion from this manuscript is that while AIE persistently impairs hippocampal neurogenesis into adulthood, despite abstinence, the cholinesterase inhibitor galantamine can both prevent and reverse these deficits. Mechanistically, results from the current study suggest that galantamine prevents and rescues AIE-induced deficits in hippocampal neurogenesis by preventing and reversing ethanol induction of pro-inflammatory cascades which then drive activation of cell death machinery in newborn neurons to regulate loss and restoration of neurogenesis.

### AIE disrupts the hippocampal environmental milieu

In the current study, we found that AIE disrupts the environmental milieu in the hippocampus toward a proinflammatory state, evidenced by increases in hippocampal CCL2, HMGB1, COX-2, and NADPH-oxidase. Our finding that AIE increases the expression of CCL2+IR is particularly exciting as recent studies have found that ethanol withdrawal acutely induces neuronal CCL2 mRNA expression in the hippocampus, among other brain regions [30, 31]. Results from the current study indicate that AIE results in a chronic induction of CCL2 in the hilus, which can have several important physiological consequences. For example, other studies have suggested that CCL2 is constitutively expressed in neurons where it is released as a neuromodulator [32], and in the hippocampus CCL2 release increases the excitability of target neurons [33] and impairs neurogenesis through actions at its receptor, CCR2, which is enriched in the neurogenic zone of the granular cell layer [26, 34]. These findings expand upon classical ideas that CCL2 proinflammatory functions are specific to glia.

Similarly, COX-2 is a proinflammatory marker which is constitutively expressed in excitatory neurons where it plays important roles in neuroplasticity as well as neuropathology [35]. In neurons, COX-2 is upregulated both in response to increased intracellular calcium following excitatory stimuli as well as downstream of more classical proinflammatory pathways, including NF-ĸB [36]. Like CCL2, COX-2 mRNA is induced during acute ethanol withdrawal [37]. In the current study, chronic induction of COX-2 may be a downstream indicator of NF-ĸB activation, which has previously been reported to be increased after AIE [10]. This induction of COX-2 after AIE may be associated with neuronal loss, including loss of neurogenesis, as its induction is similarly seen in other models of hippocampal pathology, including epilepsy, and blocking COX-2 induction can rescue this cellular damage [38]. Alternatively, induction of COX-2 could reflect a more general state of hyperexcitability within the dentate gyrus after AIE, potentially through CCL2 mediation, which has been shown to increase neuronal excitability in the hippocampus [33]. Other studies have found that AIE enhances excitability of regions downstream from the dentate gyrus circuitry, mainly CA1 [39], which could collectively suggest that AIE’s induction of a proinflammatory environment within the hippocampus has direct consequences for the physiology and excitability of neurons within hippocampal circuits which extends beyond neurogenesis.

In contrast, HMGB1+IR is densely expressed within the granular cells and as well as specifically expressed in the nucleus of DCX+ cells in the neurogenic zone. The role of HMGB1 on cell physiology is complex and largely dependent on its location within the intracellular and extracellular environments. For example, HMGB1 can play an important role in neurogenesis through modulation of the DNA transcription when expressed in the nucleus [40]. Conversely, HMGB1 can be passively or actively secreted from the cell whereupon it can activate toll-like receptors and enhance other proinflammatory cascades, including phosphorylated NF-ĸB p65, NADPH-oxidase, and COX-2 [41, 42]. The complex and interwoven nature of these proinflammatory intra- and extracellular signaling cascades suggests that AIE disrupts the overarching proinflammatory environmental milieu within the hippocampus, which has important consequences for hippocampal physiology and neurogenesis.

### Galantamine exhibits anti-inflammatory actions to prevent and reverse AIE reductions in neurogenesis

In AIE, induction of hippocampal proinflammatory markers is paralleled by a reduction of adult neurogenesis (DCX). These results expand upon an emerging body of literature which suggests that either preventing this proinflammatory induction or restoring the proinflammatory balance in the neurogenic niche after AIE results in restoration of adult neurogenesis [10]. While we have previously reported that voluntary exercise can prevent or reverse neurogenic deficits after AIE [10], the current study expands on these findings by suggesting that the cholinergic system may be an important mediator of these pathogenic cascades following AIE. This hypothesis emerged after evidence that septohippocampal cholinergic projection neurons are decreased after AIE [4], suggesting that chronic neuroimmune induction after AIE may be mediated by loss of cholinergic neuroinflammatory control. In support of this hypothesis, we report here that AIE reduces DCX+IR in adult (P70+) rats in addition to increasing proinflammatory signals, and that these effects are both prevented and reversed by galantamine.

**Fig. 10:**
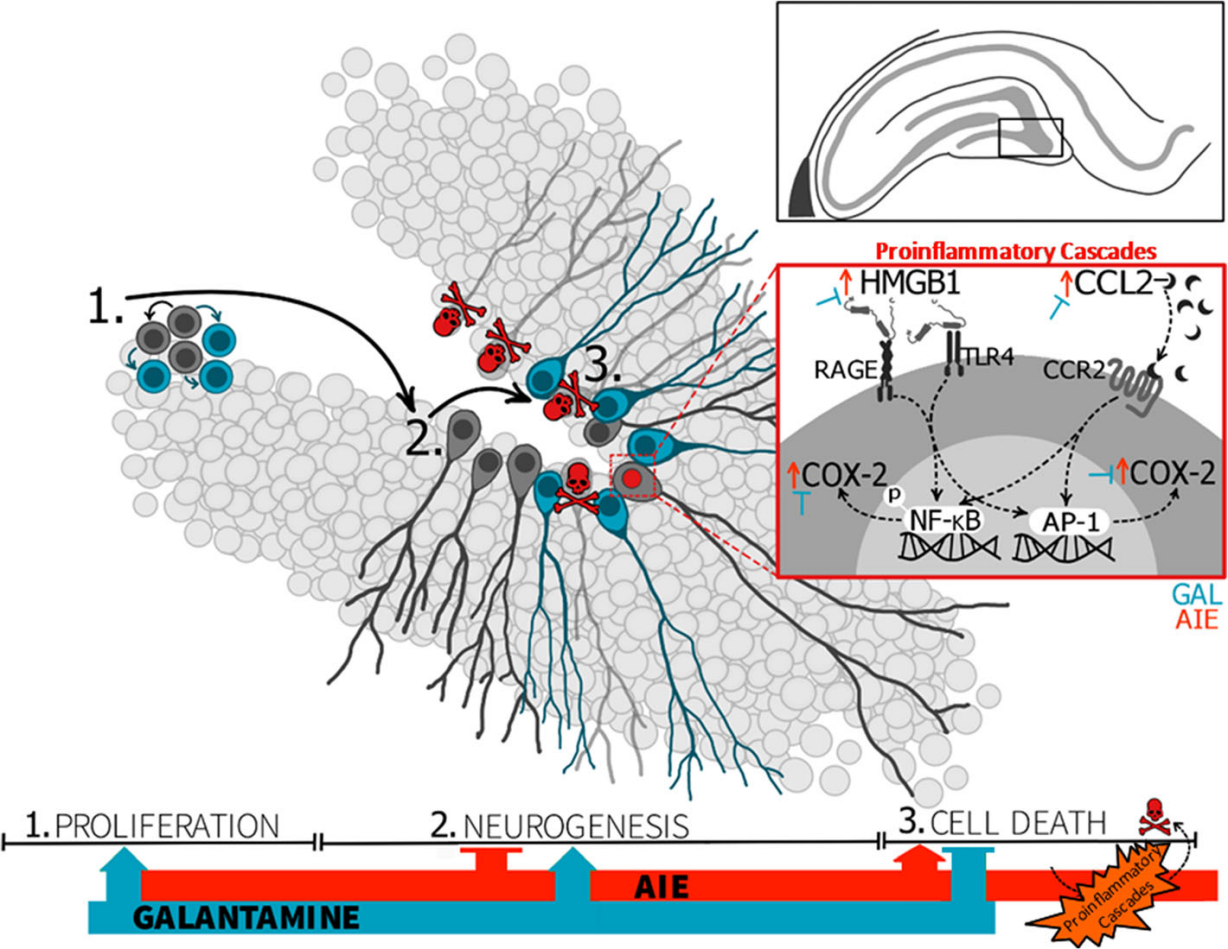
Mechanisms underlying AIE-induced hippocampal pathology. Progenitor cells in the subgranular zone of the hippocampus go through a multiweek maturation where they (1) proliferate and (2) differentiate and develop into immature neurons, during which they functionally integrate within hippocampal circuitry. Damaged or otherwise deficient cells do not survive this process, and activation of cell-apoptotic pathways is an important normal physiological checkpoint (3). However, AIE increases cell death beyond normal apoptotic levels (3), as evidenced by increases in immunoreactivity of cleaved Casp3 in immature neurons. While these neuroimmune changes are persistent despite abstinence, they are not permanent and can be prevented and reversed by pharmacological intervention with galantamine. More specifically, galantamine rescues AIE-induced deficits in hippocampal neurogenesis (2) by driving increases in cell proliferation (1) and preventing overactivation of cell death pathways in newborn neurons (3). AIE-induced proinflammatory cascades is a critical mechanism underlying AIE-induced neurogenic deficits as increased proinflammatory signaling upsets the environmental milieu necessary for healthy levels of hippocampal neurogenesis. Specifically, AIE increases HMGB1 expression, which we have previously shown activates RAGE and TLR4 receptors (as reviewed by [47]). RAGE and TLR4 signaling cascades induce phosphorylation of the transcription factors NF-ĸB and AP-1, ultimately resulting in downstream induction of other proinflammatory signaling, including COX-2. Conversely, the chemokine CCL2 is expressed in the hilus and released as a neuromodulator to activate CCR2 receptors. CCR2 receptors are expressed in the subgranular zone of the dentate where they have also been shown to influence downstream proinflammatory cascades including NF-ĸB and AP-1 phosphorylation and COX-2 signaling. These pathways create a network of positive feedback for proinflammatory signaling cascades after AIE, which are prevented and restored by galantamine

Galantamine is an FDA-approved cholinesterase inhibitor used in the treatment of Alzheimer’s disease [43]. Its anti-inflammatory effects are thought to be mediated indirectly through increased acetylcholine availability in addition to functioning as a direct allosteric potentiating ligand for nicotinic α7 as well as α4β2 receptors on neurons and glia [44]. In vivo, galantamine also directly stimulates vagal efferents which could contribute to cholinergic anti-inflammatory effects [45, 46]. In the current study, CCL2, HMGB1, and COX-2 immunoreactivity were all induced by AIE and then prevented or reversed by galantamine, suggesting that a loss of cholinergic signaling plays an important role in persistent induction of neuroinflammation by AIE (see Fig. 10). Mechanistically, acetylcholine activation of nAChRα7 blocks nuclear translocation of NF-ĸB and prevents HMGB1 release by blocking its translocation from the nucleus [48], which could suggest that galantamine’s anti-inflammatory actions may be mediated by increased nAChRα7 signaling.

However, galantamine did not reverse all of AIE’s proinflammatory effects—most notably, galantamine did not reverse AIE induction of superoxide generating NADPH (gp91^phox^). This dissociation of effects could reflect differences in cellular localization of neuroinflammatory markers. For instance, while all markers in the current study have been evidenced in both neurons and glia, histological counts of COX-2, HMGB1, and CCL2 likely reflect neuronal upregulation of these factors due to the large cellular size and shape. Conversely, western blot analyses do not allow separation of neurons and glia, and although gp91^phox^ is expressed in a small extent in neurons, it is predominantly expressed in microglia in relation to microglial regulation of phagocytosis and neuroinflammatory-induced cell loss in neurodegenerative diseases [49]. Further studies will need to examine whether distinctions between reversibility of proinflammatory responses following AIE reflect differences in specific cellular subtypes, as not all cells may exhibit similar levels of plasticity following AIE.

### Galantamine and AIE differentially impact Ki67, PCNA, and cleaved Casp3 in the neurogenic niche

Previous studies have found that AIE increases markers of cell death in the hippocampus, most notably cleaved Casp3 [12]. The current study expanded on these prior findings, indicating that AIE specifically increases activation of cell death cascades in immature neurons. These novel findings suggest that activation of cell death pathways in immature neurons is a critical mediator of AIE-induced loss of neurogenesis. Moreover, as galantamine reversed both the induction of proinflammatory cascades within the hippocampus as well as activation of cell death cascades in immature neurons after AIE, these results add to a growing body of literature which suggests a critical mechanistic link between neuroinflammation, neurodegeneration, and neurogenesis.

Conversely, galantamine also increases markers of cell proliferation, independently from ethanol exposure. This increase in cell proliferation is indicated by an increase in the number of cells expressing the proliferation marker Ki67, but not an overall increase in clusters of Ki67 cells, suggesting that galantamine may be increasing the time that actively proliferating cells are dividing as opposed to shifting non-active progenitor cells toward a state of active proliferation. These findings support results from prior studies which have also demonstrated that galantamine acutely increases cell proliferation, determined using BrdU proliferation and neuronal survival assays [50, 51]. However, our results are distinct from another cholinesterase inhibitor, donepezil, which restores hippocampal neurogenesis after AIE following acute administration through mechanisms independent of cell proliferation [12]. Interestingly, other studies which have compared the mechanisms of galantamine to donepezil have similarly found that galantamine, unlike donepezil, drives cell proliferation [51].

Surprisingly, results indicate that PCNA, which is expressed both during cell proliferation and cell repair, was not increased by galantamine. During proliferation, PCNA acts as a “sliding clamp” to recruit and hold proteins to DNA, including DNA polymerase [52]. However, PCNA is also a critical mediator of post-replication repair (PRR) pathways, where it prevents the faltering of DNA replication in order to avoid double strand breaks [53, 54]. As such, a loss of PCNA in the absence of decreased Ki67 could indicate reductions in appropriate initiation of PRR pathways during cell replication and thus an increase in likelihood of double strand breaks which lead to cell death. As AIE also increased activation of cell death pathways (cleaved Casp3) in DCX-expressing cells, this could indicate that PCNA fails to appropriately facilitate PRR after AIE, increasing the likelihood of double strand breaks in newly dividing cells and consequently increasing cell death in the neurogenic niche. However, this hypothesis requires further investigation.

Another critical mediator underlying cell proliferation, differentiation, and survival is trophic support. In the current study, we investigated two independent mechanisms of trophic support: levels of BDNF in conjunction to pan-Trk receptors (which includes its receptor, TrkB) as well as insulin-signaling trophic support with IGF2 and IRβ expression in the hippocampus. The role of BDNF in stimulating hippocampal neurogenesis is well-established (for review see [55]). Similarly, insulin signaling is a critical mediator of neurogenesis [56] and hippocampal plasticity (for review see [57]), and one of the primary mechanisms by which galantamine is thought to improve hippocampal physiology is through alleviating insulin resistance [50, 58]. While some studies have indicated that galantamine does acutely increase IGF2 [50], our findings rather indicate that these effects are not sustained long-term. Neither pro- nor mature BDNF were altered by either AIE or galantamine in the current study. These results are congruent with other studies showing that galantamine does not impact BDNF mRNA levels [50], as well as studies suggesting that BDNF levels are not changed in the dentate after AIE [59]. However, galantamine did increase hippocampal pan-Trk expression specifically in AIE-treated animals, suggesting that galantamine may selectively restore trophic signaling in AIE-treated animals by increasing receptor expression. Further studies will need to determine whether increases in pan-Trk expression are specific to the subgranular zone or rather reflective of overarching increases across the hippocampus. As such, results from the current study suggest that chronic overarching loss of trophic factor support is not likely to be a driving factor in AIE-induced loss of hippocampal neurogenesis. Instead, our studies suggest that loss of neurogenesis after AIE is mediated by persistent induction of proinflammatory cascades which drive activation of cell death machinery.

## Conclusions

Intermittent ethanol exposure across adolescence potently and persistently inhibits hippocampal neurogenesis, and these effects are associated with shifts within the neurogenic milieu toward an environment characterized by elevations in proinflammatory signaling, increased activation of cell death cascades in immature neurons, and loss of cholinergic regulation. Results from the current study (1) advance our understanding of the underlying factors driving these interconnected pathogenic cascades following AIE, and (2) provide insight into molecular targets which are necessary to reverse deficits in hippocampal pathology induced by AIE. Importantly, results from the current study highlight that acetylcholine is the fulcrum for many of these molecular events, as galantamine prevents and reverses AIE induction of neuroinflammation and reduction of neurogenesis within the hippocampus.

## Supplementary Information

**Additional file 1: Supplementary Table 1.** Summary of ANOVA Results and Post-hoc Comparisons.

**Additional file 2: Supplemental Figure 1.** Negative controls for anti-mouse and anti-rabbit primary antibodies. To control for non-specific staining of primary antibodies, negative controls were performed where the primary antibody was omitted for anti-mouse and anti-rabbit primary antibodies. Pictures were taken at a 4X magnification. **(a)** Negative control for all anti-rabbit primary antibodies. **(b)** Negative control for all anti-mouse primary antibodies.

**Additional file 3: Supplemental Figure 2.** Immunoblots for galantamine restoration study. These immunoblots include examples for gp91^phox^, IRβ, IGF2, BDNF, and pan-Trk, respectively. Groups were counterbalanced across lanes, within and between each of the four gels whenever possible. The respective group identification for each lane has been specified on every gel. Every gel includes at least two lanes with the Chameleon® Duo Protein Ladder to ensure accurate identification of bands at their respective molecular weights.

**Additional file 4: Supplemental Figure 3.** Example co-immunofluorescence of CCL2 with NeuN and GFAP in controls. The chemokine CCL2 is robustly expressed throughout the hilus. To further characterize which cell types immunostaining of CCL2 is implicated in, we performed co-label immunofluorescence for CCL2 with both NeuN and GFAP in control tissue. Scale bars (white) represent 100 μM. **(a)** Photomicrograph of co-label immunofluorescence with CCL2 (green) and NeuN (red). **(b)** Closer imaging of highlighted box in 1A reveals that CCL2 is clustered along the periphery of the soma in neurons, labeled by NeUN. **(c)** Photomicrograph of co-label immunofluorescence with CCL2 (green) and GFAP (red). **(d)** Closer imaging of the highlighted box in 1C reveals that although there is some small overlap between CCL2 and GFAP, the vast majority of hilar CCL2 is neuronal, which reflects the large cell bodies evidenced in between the astrocyte labeling. The pattern of CCL2 immunofluorescence co-localization with NeUN and GFAP highlights that the large cell bodies quantified for IHC which demonstrated an increase in CCL2+IR after AIE likely reflect neuronal induction.

## Data Availability

The datasets used and/or analyzed during the current study are available from the corresponding author on reasonable request.

## Abbreviations

AIE: Adolescent intermittent ethanol
ANOVA: Analysis of variance
BDNF: Brain-derived neurotrophic factor
BEC: Blood ethanol content
BRDU: Bromodeoxyuridine
CASP3: Caspase-3
CCL2: Chemokine (C-C motif) ligand 2
CNS: Central nervous system
COX-2: Cyclooxygenase-2
CON: Control
DCX: Doublecortin
GAL: Galantamine
HMGB1: High mobility group box 1
IGF2: Insulin-like growth factor 2
IR: Immunoreactivity
IRβ: Insulin receptor beta
NF-κB: Nuclear factor kappa-light-chain enhancer of activated B cells
Pan-Trk: Tropomyosin receptor kinase, A, B, and C proteins
PCNA: Proliferating cell nuclear antigen
PI: Protease inhibitor
RAGE: Receptor for advanced glycation end products
VEH: Vehicle
