# Supplementary material for: Galantamine prevents and reverses neuroimmune induction and loss of adult hippocampal neurogenesis following adolescent alcohol exposure
Source: J Neuroinflammation. 2021 Sep 16;18:212. doi: 10.1186/s12974-021-02243-7 (PMC8447570; doi:10.1186/s12974-021-02243-7)
Supplement: Supplementary file 1 — Additional file 1: Supplementary Table 1. Summary of ANOVA Results and Post-hoc Comparisons. [file 12974_2021_2243_MOESM1_ESM.docx]

*Supplementary Table 1. Summary of ANOVA Results and Post-hoc Comparisons*

| **Study** | **Dependent Variable** | **Comparison** | **df** | ***F-*value** | ***p*-values** | **Missing Data/Outliers** |
| --- | --- | --- | --- | --- | --- | --- |
| Prevention (1) | DCX | AIE × Rx  Post hoc  CON-Veh × AIE-Veh  CON-Gal × AIE-Gal  CON-Veh × CON-Gal  AIE-Veh × AIE-Gal | 1, 34 | 8.45 | 0.006  0.006  0.285  0.445  < 0.001 | 4 |
|  | Ki67 | AIE × Rx  Rx | 1, 38  1, 38 | 0.94  14.09 | 0.339  0.001 | 0 |
|  | PCNA | AIE × Rx  Post hoc  CON-Veh × AIE-Veh  CON-Gal × AIE-Gal  CON-Veh × CON-Gal  AIE-Veh × AIE-Gal | 1, 38 | 4.72 | 0.036  0.001  0.422  0.128  0.138 | 0 |
|  | Cleaved Casp3/DCX  co-localized | AIE × Rx  Post hoc  CON-Veh × AIE-Veh  CON-Gal × AIE-Gal  CON-Veh × CON-Gal  AIE-Veh × AIE-Gal | 1, 38 | 23.33 | < 0.001  < 0.001  0.018  0.064  < 0.019 | 0 |
|  | HMGB1 | AIE × Rx  Post hoc  CON-Veh × AIE-Veh  CON-Gal × AIE-Gal  CON-Veh × CON-Gal  AIE-Veh × AIE-Gal | 1, 38 | 3.89 | 0.060  0.036  0.662  0.138  0.013 | 0 |
|  | COX-2 | AIE  Post hoc  CON-Veh × AIE-Veh  CON-Gal × AIE-Gal  CON-Veh × CON-Gal  AIE-Veh × AIE-Gal | 1, 38 | 6.44 | 0.015  0.043  0.153  0.766  0.243 | 0 |
|  | CCL2 | AIE × Rx  Post hoc  CON-Veh × AIE-Veh  CON-Gal × AIE-Gal  CON-Veh × CON-Gal  AIE-Veh × AIE-Gal | 1, 38 | 16.91 | < 0.001  < 0.001  0.470  0.562  < 0.001 | 0 |
| Restoration (2) | DCX | AIE × Rx  Post hoc  CON-Veh × AIE-Veh  CON-Gal × AIE-Gal  CON-Veh × CON-Gal  AIE-Veh × AIE-Gal | 1, 35 | 8.90 | 0.005  0.001  0.587  0.080  0.021 | 0 |
|  | Ki67 | AIE × Rx  Rx | 1, 34  1, 34 | 3.78  21.51 | 0.06  < 0.001 | 1 |
|  | PCNA | AIE × Rx  Post hoc  CON-Veh × AIE-Veh  CON-Gal × AIE-Gal  CON-Veh × CON-Gal  AIE-Veh × AIE-Gal | 1, 35 | 3.71 | 0.060  0.069  0.412  0.097  0.312 | 0 |
|  | Cleaved Casp3/DCX  co-localized | AIE  Post hoc  CON-Veh × AIE-Veh  CON-Gal × AIE-Gal  CON-Veh × CON-Gal  AIE-Veh × AIE-Gal | 1, 35 | 4.43 | 0.043  < 0.001  0.010  0.064  < 0.019 | 0 |
|  | HMGB1 | AIE × Rx  Post hoc  CON-Veh × AIE-Veh  CON-Gal × AIE-Gal  CON-Veh × CON-Gal  AIE-Veh × AIE-Gal | 1, 35 | 10.59 | 0.003  < 0.001  0.731  0.525  0.027 | 0 |
|  | COX-2 | AIE × Rx  Post hoc  CON-Veh × AIE-Veh  CON-Gal × AIE-Gal  CON-Veh × CON-Gal  AIE-Veh × AIE-Gal | 1, 34 | 8.75 | 0.006  < 0.001  0.577  0.970  < 0.001 | 1 |
|  | IRβ | AIE × Rx | 1, 25 | 0.31 | 0.582 | 0 |
|  | ProIGF2 | AIE × Rx | 1, 25 | 0.07 | 0.800 | 0 |
|  | IGF2 | AIE × Rx | 1, 25 | 0.31 | 0.584 | 0 |
|  | ProBDNF | AIE × Rx | 1, 25 | 0.29 | 0.593 | 0 |
|  | Mature BDNF | AIE × Rx | 1, 25 | 0.76 | 0.393 | 0 |
|  | Pan-Trk | AIE × Rx  Post hoc  CON-Veh × AIE-Veh  CON-Gal × AIE-Gal  CON-Veh × CON-Gal  AIE-Veh × AIE-Gal | 1, 25 | 5.78 | 0.024  0.571  0.008  0.243  0.041 | 0 |
|  | gp91-phox | AIE | 1, 25 | 4.63 | 0.042 | 0 |

*Variable codes: AIE (CON, AIE); Rx (Vehicle, Galantamine). *Bonferroni corrected post hoc analyses.*
