# Supplementary figures and images for: Galantamine prevents and reverses neuroimmune induction and loss of adult hippocampal neurogenesis following adolescent alcohol exposure
Source: J Neuroinflammation. 2021 Sep 16;18:212. doi: 10.1186/s12974-021-02243-7 (PMC8447570; doi:10.1186/s12974-021-02243-7)

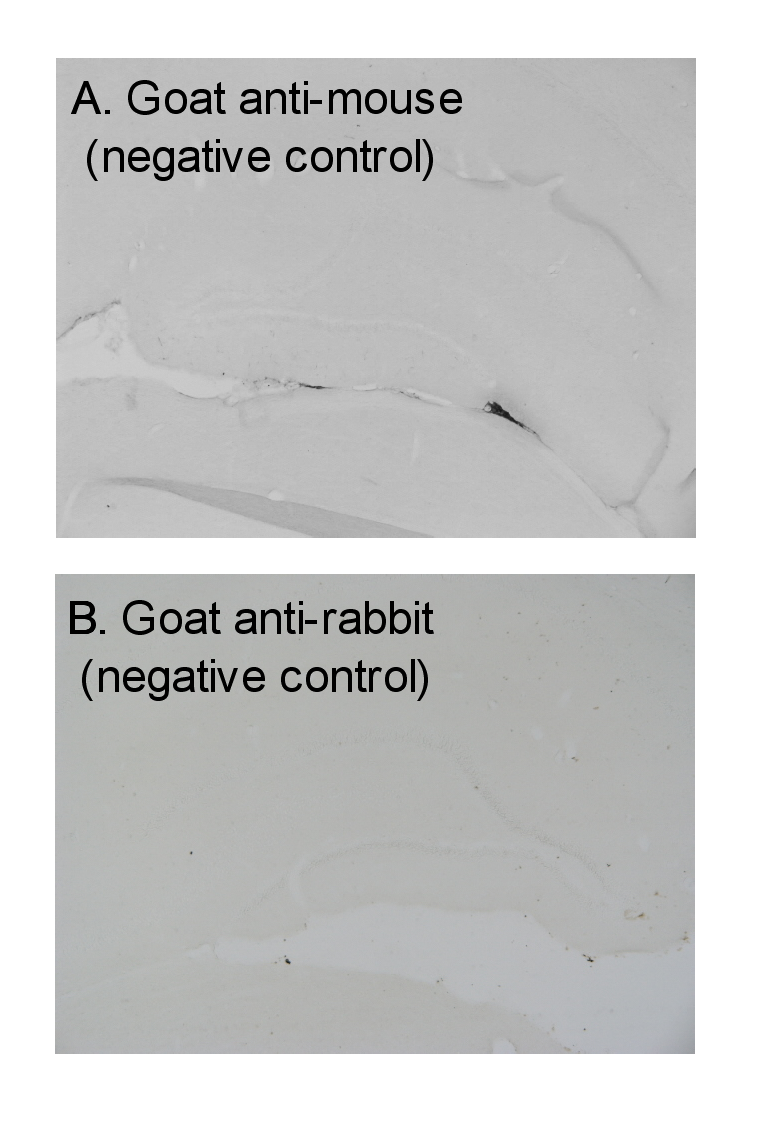

Supplement: Supplementary file 2 — Additional file 2: Supplemental Figure 1. Negative controls for anti-mouse and anti-rabbit primary antibodies. To control for non-specific staining of primary antibodies, negative controls were performed where the primary antibody was omitted for anti-mouse and anti-rabbit primary antibodies. Pictures were taken at a 4X magnification. (a) Negative control for all anti-rabbit primary antibodies. (b) Negative control for all anti-mouse primary antibodies. [file 12974_2021_2243_MOESM2_ESM.tif]

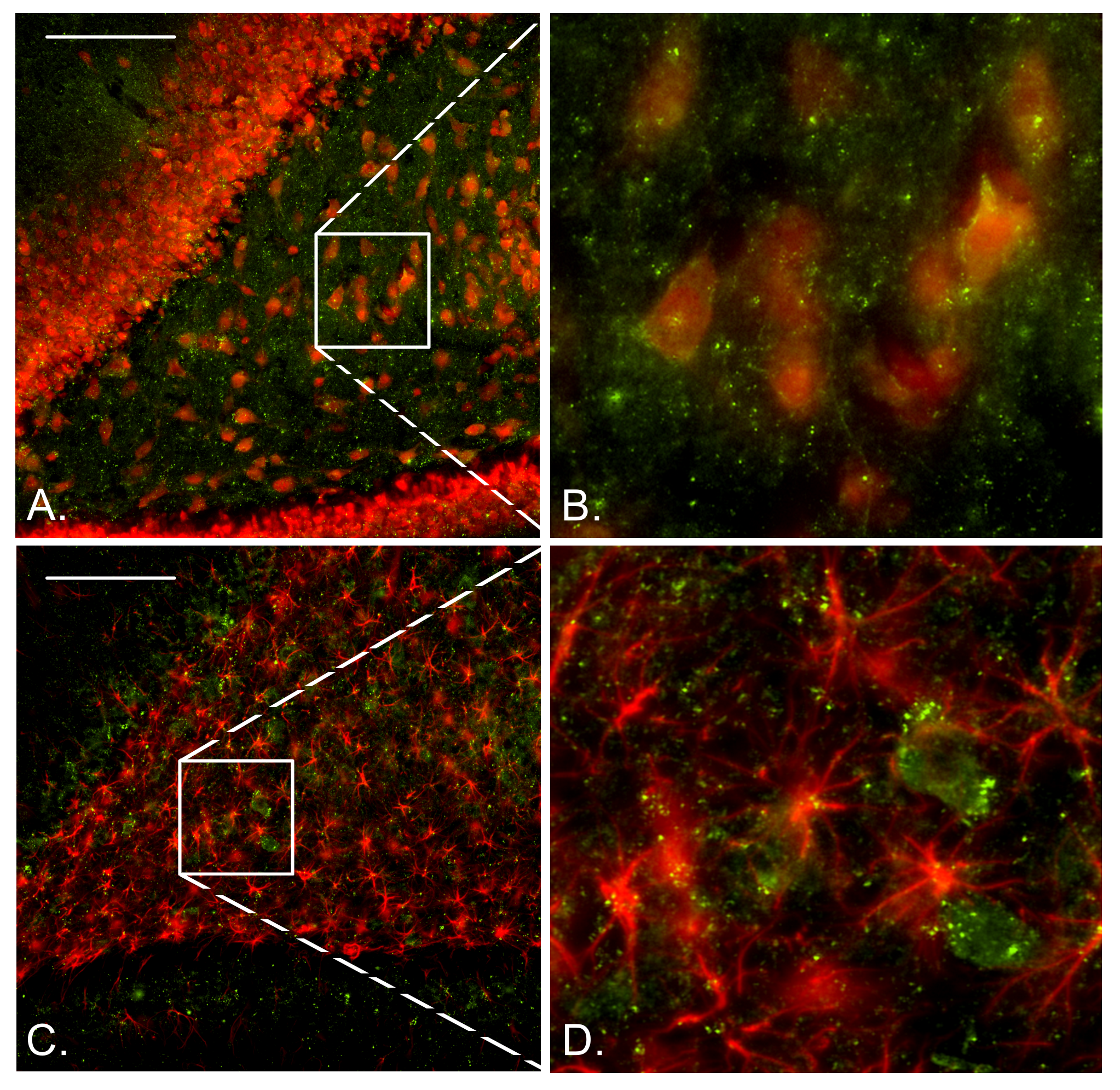

Supplement: Supplementary file 3 — Additional file 3: Supplemental Figure 2. Immunoblots for galantamine restoration study. These immunoblots include examples for gp91phox, IRβ, IGF2, BDNF, and pan-Trk, respectively. Groups were counterbalanced across lanes, within and between each of the four gels whenever possible. The respective group identification for each lane has been specified on every gel. Every gel includes at least two lanes with the Chameleon® Duo Protein Ladder to ensure accurate identification of bands at their respective molecular weights. [file 12974_2021_2243_MOESM3_ESM.tif]
