# Supplementary material for: Galantamine prevents and reverses neuroimmune induction and loss of adult hippocampal neurogenesis following adolescent alcohol exposure
Source: J Neuroinflammation. 2021 Sep 16;18:212. doi: 10.1186/s12974-021-02243-7 (PMC8447570; doi:10.1186/s12974-021-02243-7)
Supplement: Supplementary file 4 — Additional file 4: Supplemental Figure 3. Example co-immunofluorescence of CCL2 with NeuN and GFAP in controls. The chemokine CCL2 is robustly expressed throughout the hilus. To further characterize which cell types immunostaining of CCL2 is implicated in, we performed co-label immunofluorescence for CCL2 with both NeuN and GFAP in control tissue. Scale bars (white) represent 100 μM. (a) Photomicrograph of co-label immunofluorescence with CCL2 (green) and NeuN (red). (b) Closer imaging of highlighted box in 1A reveals that CCL2 is clustered along the periphery of the soma in neurons, labeled by NeUN. (c) Photomicrograph of co-label immunofluorescence with CCL2 (green) and GFAP (red). (d) Closer imaging of the highlighted box in 1C reveals that although there is some small overlap between CCL2 and GFAP, the vast majority of hilar CCL2 is neuronal, which reflects the large cell bodies evidenced in between the astrocyte labeling. The pattern of CCL2 immunofluorescence co-localization with NeUN and GFAP highlights that the large cell bodies quantified for IHC which demonstrated an increase in CCL2+IR after AIE likely reflect neuronal induction. [file 12974_2021_2243_MOESM4_ESM.pdf]

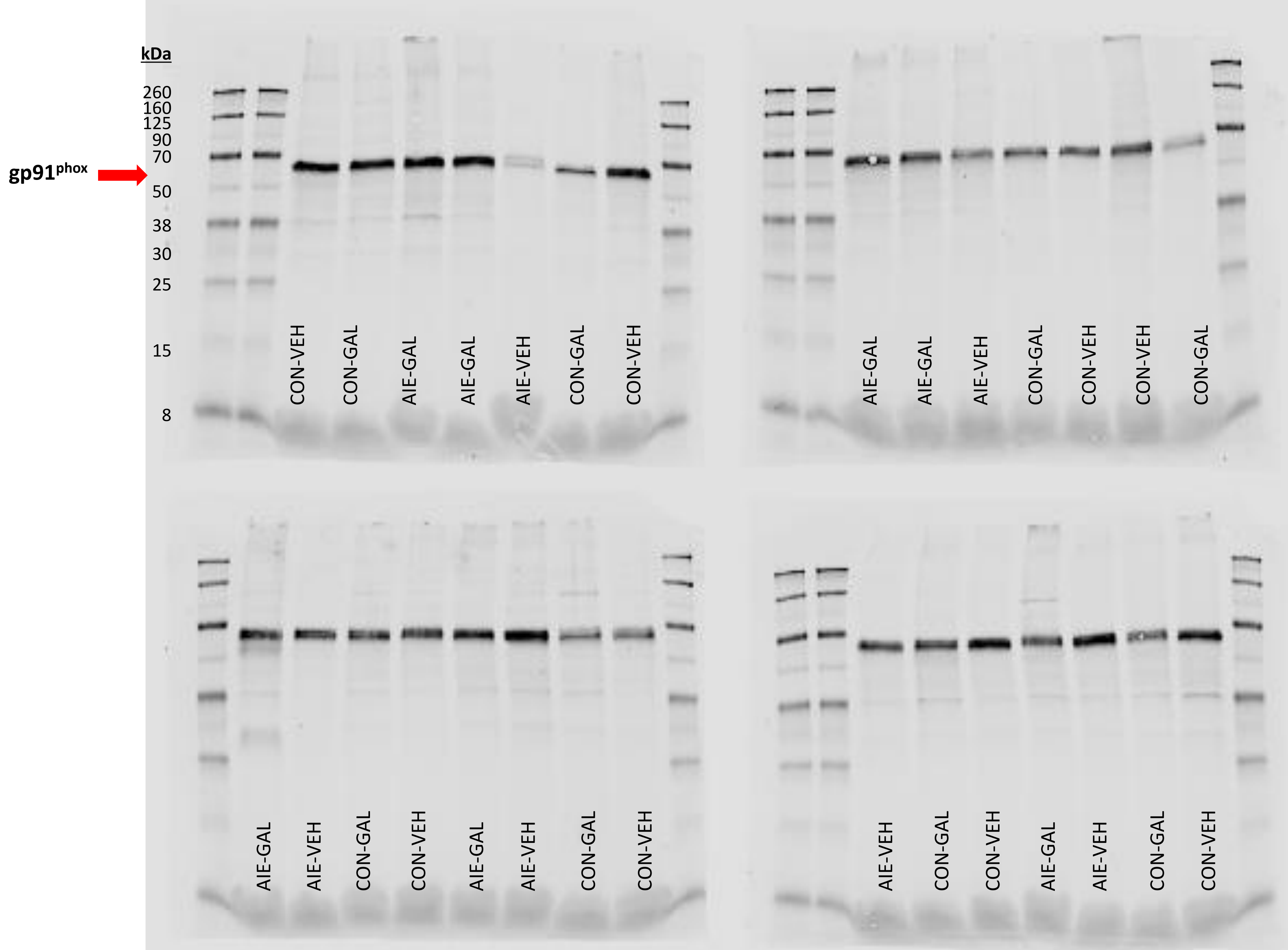
kDa
260
160
125
90
70
gp91phox
50
38
30
25
15
8
CON-VEH
CON-GAL
AIE-GAL
AIE-GAL
AIE-VEH
CON-GAL
CON-VEH
AIE-GAL
AIE-GAL
AIE-VEH
CON-GAL
CON-VEH
CON-VEH
CON-GAL
AIE-GAL
AIE-VEH
CON-GAL
CON-VEH
AIE-GAL
AIE-VEH
CON-GAL
CON-VEH
AIE-VEH
CON-GAL
CON-VEH
AIE-GAL
AIE-VEH
CON-GAL
CON-VEH

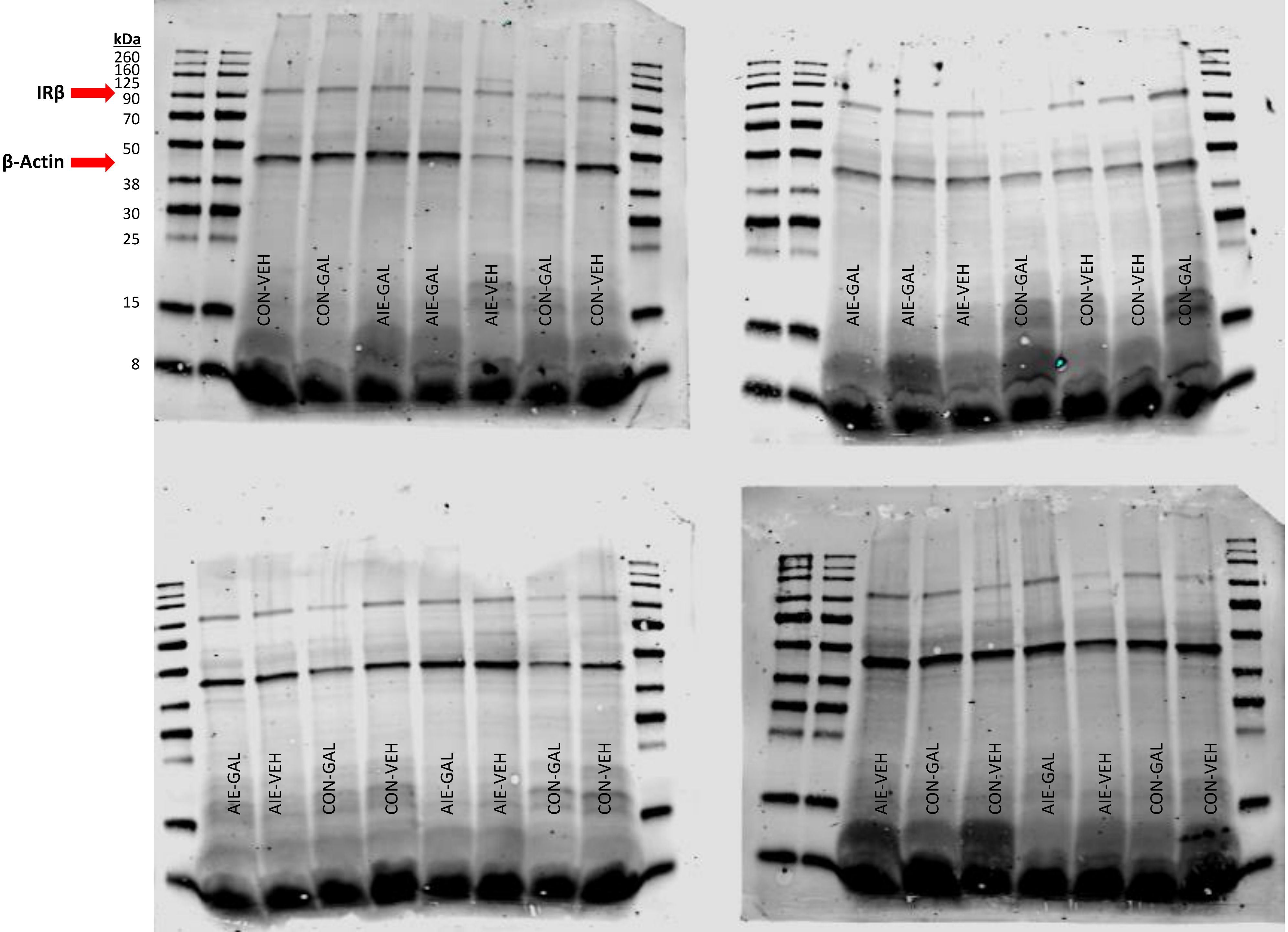

kDa
260
160
125
90
70
50
38
30
25
15
8
IRβ
β-Actin
CON-VEH
CON-GAL
AIE-GAL
AIE-GAL
AIE-VEH
CON-GAL
CON-VEH
AIE-GAL
AIE-GAL
AIE-VEH
CON-GAL
CON-VEH
CON-VEH
CON-GAL
AIE-GAL
AIE-VEH
CON-GAL
CON-VEH
AIE-GAL
AIE-VEH
CON-GAL
CON-VEH
AIE-VEH
CON-GAL
CON-VEH
AIE-GAL
AIE-VEH
CON-GAL
CON-VEH

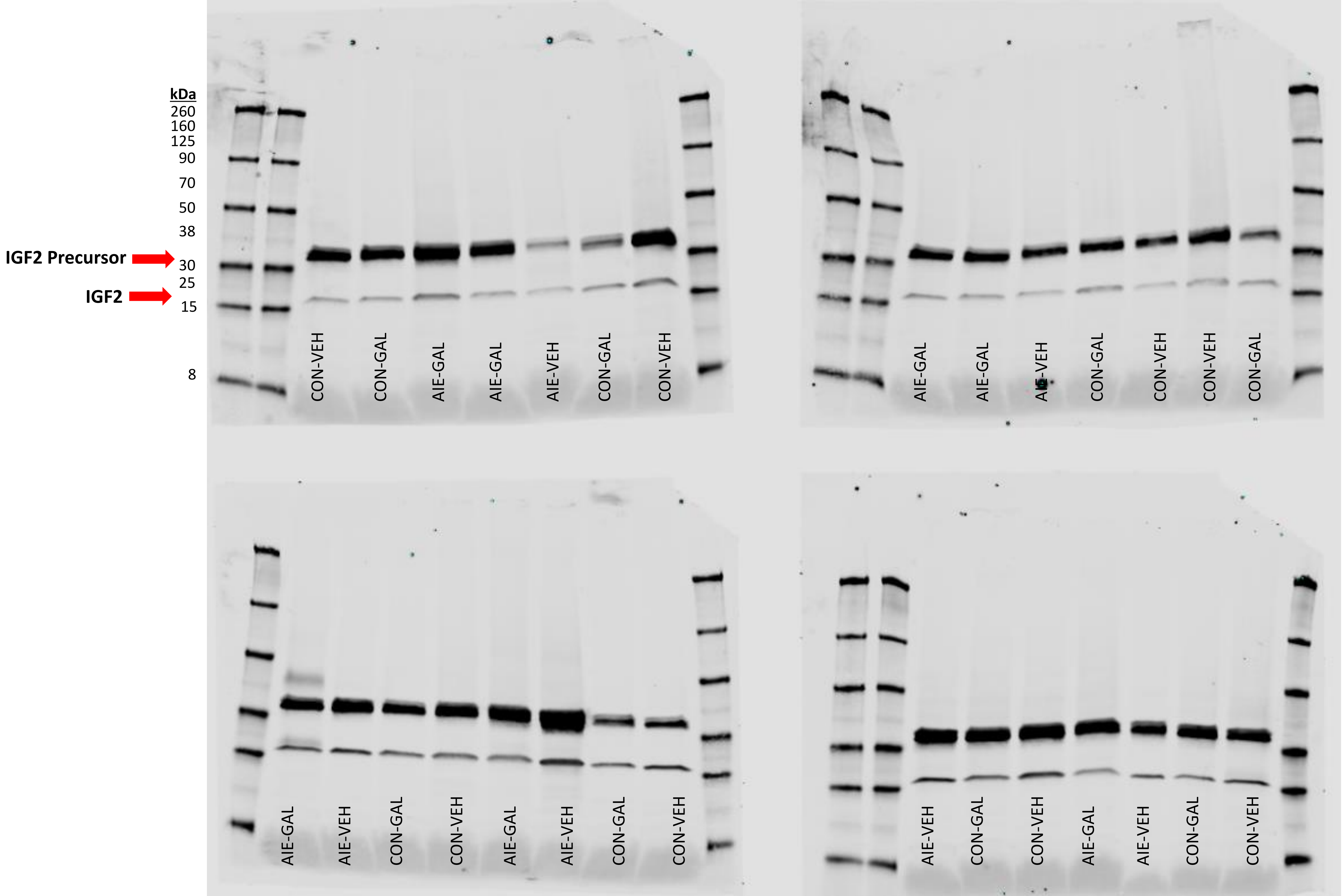
kDa
260
160
125
90
70
50
38
IGF2 Precursor
30
25
IGF2
15
8
CON-VEH
CON-GAL
AIE-GAL
AIE-GAL
AIE-VEH
CON-GAL
CON-VEH
AIE-GAL
AIE-GAL
AIE-VEH
CON-GAL
CON-VEH
CON-VEH
CON-GAL
AIE-GAL
AIE-VEH
CON-GAL
CON-VEH
AIE-GAL
AIE-VEH
CON-GAL
CON-VEH
AIE-VEH
CON-GAL
CON-VEH
AIE-GAL
AIE-VEH
CON-GAL
CON-VEH

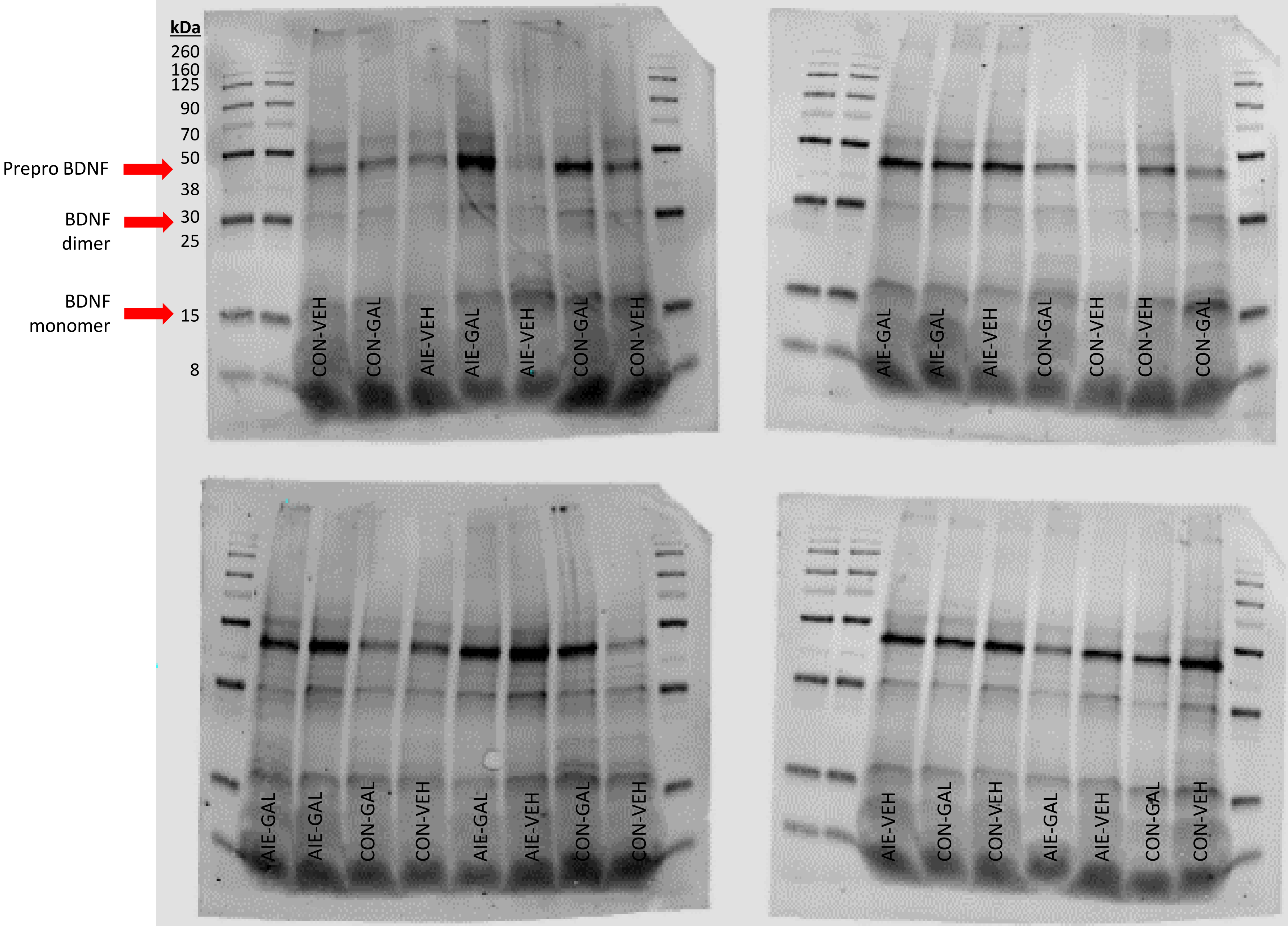

BDNF

50-65 kDa, glycosylated prepro BDNF dimer

35kDa, glycosylated prepro BDNF

28 BDNF dimer

14 kDa, mature BDNF

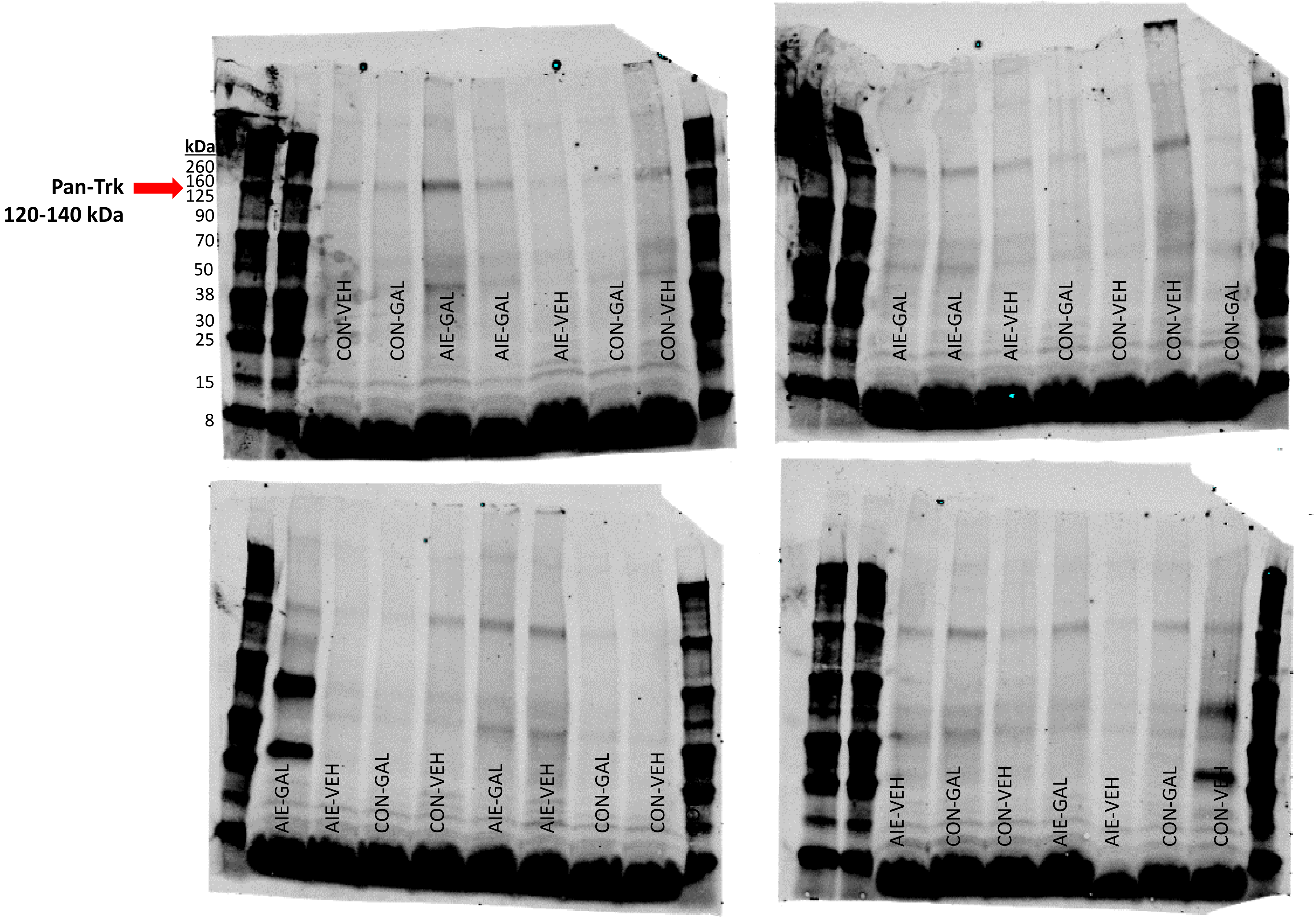
Pan-Trk
120-140 kDa
kDa
260
160
125
90
70
50
38
30
25
15
8
CON-VEH
CON-GAL
AIE-GAL
AIE-GAL
AIE-VEH
CON-GAL
CON-VEH
AIE-GAL
AIE-GAL
AIE-VEH
CON-GAL
CON-VEH
CON-VEH
CON-GAL
AIE-GAL
AIE-VEH
CON-GAL
CON-VEH
AIE-GAL
AIE-VEH
CON-GAL
CON-VEH
AIE-VEH
CON-GAL
CON-VEH
AIE-GAL
AIE-VEH
CON-GAL
CON-VEH
